# Isthmin-1 Improves Aging-Related Cardiac Dysfunction in Mice through Enhancing Glycolysis and SIRT1 Deacetylase Activity

**DOI:** 10.14336/AD.2024.0113

**Published:** 2024-01-25

**Authors:** Min Hu, Xin Zhang, Yi-Peng Gao, Yu-Xin Hu, Teng Teng, Sha-Sha Wang, Qi-Zhu Tang

**Affiliations:** ^1^Department of Cardiology, Renmin Hospital of Wuhan University, Wuhan 430060, China.; ^2^Hubei Key Laboratory of Metabolic and Chronic Diseases, Wuhan 430060, China.; ^3^Department of Geriatrics, Renmin Hospital of Wuhan University, Wuhan 430060, China

**Keywords:** Inflammation, Glycolysis, Aging, Isthmin-1, SIRT1

## Abstract

Aging-related cardiac dysfunction poses a major risk factor of mortality for elderly populations, however, efficient treatment for aging-related cardiac dysfunction is far from being known. Isthmin-1 (ISM1) is a novel adipokine that promotes glucose uptake and acts indispensable roles in restraining inflammatory and fibrosis. The present study aims to investigate the potential role and molecular mechanism of ISM1 in aging-related cardiac dysfunction. Aged and matched young mice were overexpressed or silenced with ISM1 to investigate the role of ISM1 in aging-related cardiac dysfunction. Moreover, H9C2 cells were stimulated with D-galactose (D-gal) to examine the role of ISM1 in vitro. Herein, we found that cardiac-specific overexpression of ISM1 significantly mitigated insulin resistance by promoting glucose uptake in aging mice. ISM1 overexpression alleviated while ISM1 silencing deteriorated cellular senescence, cardiac inflammation, and dysfunction in natural and accelerated cardiac aging. Mechanistically, ISM1 promoted glycolysis and activated Sirtuin-1 (SIRT1) through increasing glucose uptake. ISM1 increased glucose uptake via translocating GLUT4 to the surface, thereby enhancing glycolytic flux and hexosamine biosynthetic pathway (HBP) flux, ultimately leading to increased SIRT1 activity through O-GlcNAc modification. ISM1 may serve as a novel potential therapeutic target for preventing aging-related cardiac disease in elderly populations. ISM1 prevents aging-related cardiac dysfunction by promoting glycolysis and enhancing SIRT1 deacetylase activity, making it a promising therapeutic target for aging-related cardiac disease.

## INTRODUCTION

Aging is an important risk factor for cardiovascular diseases, and aging-related cardiac events figure negative impacts on quality of live and increase the mortality in the elderly [1]. The aging heart exhibits impaired metabolic flexibility with a decreased capacity to oxidize fatty acids and enhanced dependence on glucose metabolism, which in line with pathophysiological changes such as cardiac hypertrophy and impaired contractile function [2]. Aging-related cardiac dysfunction is associated with insulin resistance and glucose intolerance [3-6]. Besides, defective glucose metabolism in the aging heart is correlated with inflammation [7]. Glucose, as a vital metabolic substrate for the heart, plays a crucial role in various types of heart failure. Within cardiac myocytes, glucose is initially phosphorylated to glucose 6-phosphate and subsequently metabolized through multiple metabolic pathways, including glycolysis and the hexosamine biosynthetic pathway [8]. Increased glucose uptake has been implicated in various cardiovascular diseases (e.g., cardiac hypertrophy, heart failure, ischemia and aging) [9]. Tian et al. identified that increased myocardial glucose uptake prevented the development of heart failure and improved the survival of mice subjected to chronic pressure overload [10]. Additionally, increasing glucose delivery plays cardioprotective during ischemic injury [11, 12]. Increased glucose uptake also is beneficial to aging heart via enhancing generation of ATP from glycolysis [13]. Therefore, increasing glucose metabolism may be of great therapeutic interest to treat aging-related cardiac dysfunction.

SIRT1 is a NAD-dependent deacetylase, involved in the regulation of senescence through the acetylation/deacetylation of downstream substrates, and has been suggested as a key role in aging-related cardiac dysfunction [14, 15]. Studies verified that activation of SIRT1 inhibits the production of proinflammatory mediators and exerts anti-hypertensive effect in aging hearts [16, 17]. Our previous studies proved that SIRT1 activation could alleviate doxorubicin-, isoproterenol-induced or obesity-related cardiac dysfunction [18-20]. Therefore, the modulation of SIRT1 activity may play an important role as a promising therapeutic candidate for aging-related cardiac inflammation and dysfunction.

ISM1, a secreted protein is closely related to glucose metabolism and could promote glucose uptake by translocating GLUT4 to the cell surface [21]. Of note, GLUT4 can be impaired during aging, reducing glucose uptake and utilization in cardiomyocytes [22]. Ge et al. recently verified that ISM1 could restrain inflammatory through suppressing NF-κB activation and pro-inflammatory cytokine/chemokine production [23]. Additionally, administration recombinant ISM1 (rISM1) could preserve pulmonary inflammation and fibrosis in mice [23, 24]. However, whether ISM1 could ameliorate aging-induced cardiac dysfunction by improving glucose metabolism is unknown. Here, we aim to uncover the role of ISM1 in aging-related cardiac dysfunction and explore the underlying mechanisms.

## MATERIALS AND METHODS

### Reagents

The assay kits to detect serum triglyceride (TG, A110-2-1)and total cholesterol (TC, A111-2-1) were obtained from Nanjing Jiancheng Bioengineering Institute (Nanjing, China). Senescence-Associated β-Galactosidase (SA-β gal) Staining Kit (#9860) was purchased from Cell Signaling Technology (Danvers, Massachusetts, USA). The IL-1β, IL-6, IL-18, and TNF-α were purchased from Thermo Fisher Scientific (Waltham, MA, USA). SIRT1 Activity Assay Kit (ab156065) and Caspase-1 Assay Kit (ab39412) were purchased from Abcam (Cambridge, UK). lucigenin (#M8010), glucosamine hydrochloride (GlcN, #G4875), 6-diazo-5-oxo-L-norleucine (DON, #D2141), alloxan monohydrate (ALX, #A7413), thiamet G (TMG, #SML0244) and AKT inhibitor (AKT i, #124005), D-galactose (#G5388) were purchased from Sigma-Aldrich (St. Louis, MO, USA). EnzyChrom™ Glycogen Assy Kit (E2GN-100) was purchased from BioAssay Systems (Hayward, CA, USA). The goat anti-mouse IgG Alexa Fluor 488 secondary antibodies (#A11001), and SlowFade™ gold antifade reagent with DAPI (#S36939), Click-iT protein analysis detection kit (#C33372) and Click-iT O-GlcNAc enzymatic labeling kit (#C33368) were purchased from Invitrogen (Carlsbad, CA, USA). Mouse ISM1 ELISA Kit (#MBS 9355419) and human ISM1 ELISA Kit (#MBS 2707255) were purchased from Mybiosource (San Diego, CA, USA). ATP Assay Kit (S0026) was purchased from Beyotime (Shanghai, China). Adeno associated virus serotype 9 (AAV9) carrying human ISM1 under the control of a cTnT promoter (AAV9-hISM1) or a negative control (AAV9-Ctrl) were obtained from DesignGene Biotechnology (Shanghai, China). Two independent short hairpin RNAs against mouse *Ism1* (sh*Ism1* and sh*Ism1*#) or a control sh*NC* carried by AAV9 vectors under the cTnT promoter were used to specifically silence ISM1 in murine hearts. Adenovirus carrying human ISM1 (AdhISM1) or a control AdNC were obtained from Hanbio Biotechnology (Shanghai, China). Small interfering RNA against rat ISM1 (si*Ism1* and si*Ism1*#) or scramble si*NC* were all obtained from RiboBio Biotechnology (Guangzhou, China).

### Animals and treatments

All animal experiments were approved by the Animal Care and Use Committee of Renmin Hospital of Wuhan University and performed in compliance with the *Guidelines for Care and Use of Laboratory Animals* published by the US National Institutes of Health (NIH Publication No. 85-23, revised 1996) and permitted by the Animal Care and Use Committee of the Wuhan University Renmin Hospital (IvD number: WDRM 20230509A). Male C57BL/6 mice were purchased from the Institute of Laboratory Animal Science, Chinese Academy of Medical Sciences, and housed in an environment-controlled SPF barrier system with free access to food and water. After acclimation for one week, 6-month (M)-old young and matched 18-M-Old aging mice were injected with 1 × 10^11^ viral genome AAV9-hISM1 or AAV9-sh*Ism1* per mouse from the tail vein to specifically overexpress or silence ISM1 in the myocardium. Eight weeks after AAV9 injection, mice were subjected to cardiac functional measurements and then sacrificed with the heart and serum samples collected for further investigation. The accelerated cardiac aging was induced by D-gal treatment, which is a well-established aging model in vitro and in vivo [25]. 8 weeks mice were subcutaneous injection with D-gal (150 mg/kg daily) [26]. To verify the role of SIRT1, *Sirt1* cardiac knockout (*Sirt1-cKO*) mice were used according to our previous studies [18].

### Echocardiography and hemodynamics

Echocardiography and hemodynamics were performed as we previously described [27, 28]. Briefly, Mice were placed on a preheated pad and anesthetized with 1.5% isoflurane to provide adequate sedation. Vevo® 3100 High-Resolution Preclinical Imaging System (FUJIFILM VisualSonics) was used to record functional parameters. Invasive hemodynamic parameters were collected using a 1.4F Millar catheter transducer (SPR-839; Millar Instruments) and analyzed by the PVAN data analysis software.

### Histological analysis

Cardiomyocyte cross-sectional area and collagen deposition were determined by Masson's trichrome (collagen, blue; cytoplasm, red/pink) or Wheat Germ Agglutinin (WGA) staining according to our previously reported [29].

### Cells and treatments

H9C2 cells were regularly cultured in high-glucose DMEM containing 10% fetal bovine serum, 100 U/ml penicillin and 100 mg/ml streptomycin at 37°C in a humidified incubator under 5% CO2.To knock down endogenous *Ism1* in vitro, H9C2 cells were pre-transfected with 50 nmol/L si*Ism1* for 4 h using a Lipo6000™ transfection reagent according to the manufacturer′s instructions, followed by the incubation in fresh medium for an additional 24 h, then, the cells were treated with D-gal for 24h [30].

### Immunofluorescence staining

Immunohistochemical staining was performed to analyze the expression of CD45 and CD68 in the myocardium as we previously described [31]. Briefly, deparaffinized sections were subjected to high-pressure antigen retrieval process, and then blocked with 3% hydrogen peroxide and 10% goat serum. For immunofluorescence staining of GLUT4, cells were fixed with 4% paraformaldehyde for 15 min and permeabilized in 1% Triton X-100 for 5 min at room temperature. To block the non-specific binding, cell coverslips were incubated with 10% goat serum. Next, cardiac slices and cell coverslips were incubated with matched antibody at 4 °C overnight and horseradish peroxidase (HRP)-conjugated secondary antibodies at 37 °C for an additional 1 h, visualized with diaminobenzidine and analyzed using the Image-Pro Plus 6.0 software.

### SA-β gal staining

SA-β gal staining was performed according to our previous study [27]. Briefly, fresh frozen heart sections or H9C2 cells were fixed with the fixation buffer at room temperature for 15 min and then incubated with the β-gal staining solution at 37°C for 24 h in a dry incubator. Then the staining cells were captured using the light microscopy, and the senescent cells were blue color, and the percentage of SA-β gal+ cells were quantified from at least 5 high-magnification fields.

### Telomere length measurement

Telomere length was measured based on a real-time PCR method as our previously described [27]. Briefly, genomic DNA was extracted from the heart samples and cells, and then, the ratio of telomere repeat copy number to the copy number of a single-gene, acidic ribosomal phosphoprotein PO forward (36B4) was calculated as the telomere length.

### Glucose uptake

Glucose uptake was measured according to previous study [21, 32]. Briefly, mice were injected I.P. with 3H-2-deoxyglucose (3H-2-DOG) at 100 mCi/kg. After 60 min, mice were euthanized, and the hearts were collected, and 20 to 40 mg of crushed cardiac tissue was homogenized in 1500 ml of dH2O. Samples were spun at 6500g for 10 min at 4°C. To determine total (3H-2-DOG) radioactivity, we counted 500 ml of the supernatant by liquid scintillation.

### Biochemical analysis

The levels of TNF-α, IL-1β, IL-18 and IL-6 in cardiac extracts were determined by commercially available ELISA kits following the standard protocols. Fasting blood glucose (FBG) was examined using a One Touch Ultra Easy glucometer (Life Scan, Wayne, PA, USA), and the levels of serum ISM1, TG, TC and the activity of caspase-1 were detected by the commercial kits according to the manufacturer’s instructions.

### Immunoprecipitation (IP)

Cell lysates were immunoprecipitated with specific antibody or control serum, followed by incubation with protein A/G beads. After washing with the lysis buffer, the co-precipitated proteins were identified by western blotting analysis.

### O-GlcNAc enzymatic labeling

Cells were lysed in RIPA buffer. Endogenous SIRT1 was immunoprecipitated from cell lysate (800 μg) using anti-SIRT1 antibody. The immunopurified SIRT1 was enzymatically labeled with an azido-containing nucleotide sugar analog (UDP-GalNAz) using an engineered (1,4)-galactosyltransferase (GalT1 Y289L) according to the Click-iT O-GlcNAc enzymatic labeling kit protocol and conjugated with an alkyne-biotin compound as per the Click-iT protein analysis detection kit protocol. Biotin-labeled samples were subsequently probed with STVHRP. Control experiments were performed in parallel in the absence of GalT1 Y289L.

### RNA-sequencing

The hearts of 20-month mice (20 M) and control mice (8 M) were subjected to RNA-sequencing. RNA was extracted, and the quality of the extracted total RNA samples was checked with RNA 6000 Nano kit of the Bioanalyzer 2100 system (Agilent Technologies, CA, USA). RNA-seq and subsequent analyses were performed by OE Biotech Co., Ltd. (Shanghai, China).

### Quantitative real-time PCR and Western blot

Total RNA was extracted using TRIzol reagent and then reversely transcribed to cDNA with a Maxima First Strand cDNA Synthesis Kit (Roche, Basel, Switzerland). Gene expression was determined by Roche LightCycler® 480 detection system using SYBR Green 1 Master Mix (Roche), and GAPDH was selected as an internal control, and the primer sets used were provided in supplementary Table 2. Protein extraction and western blot were performed according to our previous studies [33]. Briefly, total proteins were extracted from murine hearts or cells using RIPA lysis buffer, and then quantified with a BCA protein assay kit. After that, equal amounts of total proteins were electrophoresed by the SDS-PAGE and transferred onto polyvinylidene fluoride membranes. After being blocked in 5% skimmed milk at room temperature for 1 h, the membranes were probed with primary antibodies (Table S1) at 4 °C overnight. Then, the membranes were incubated with horseradish peroxidase-conjugated secondary antibodies at room temperature for an additional 1h on the next day. The protein bands were visualized by a ChemiDoc™ XRS +system and analyzed with the Image Lab software (Bio-Rad Laboratories, Inc.). Nuclear and membrane proteins were extracted by commercial kits according to the manufacturer's instructions, and Lamin B1 in nuclear fractions and GAPDH in cell lysates were used as internal controls.

### Statistical analysis

All data are expressed as the mean ± standard deviation and analyzed using GraphPad Prism (version 8.0). The Shapiro-Wilk test is used to assess the normal distribution of all data. Unpaired Student's t test was used to compare differences between two groups, while one-way analysis of variance followed by Tukey's post hoc test was performed to compare the differences among multiple groups. The Kruskal-Wallis test was used to detect significant differences for non-normal data or small sample sizes. All values are expressed as the mean ± standard deviation and p<0.05 was considered significant.

## RESULTS

### ISM1 modulates D-gal-induced cellular senescence in H9C2

**Figure 1.**
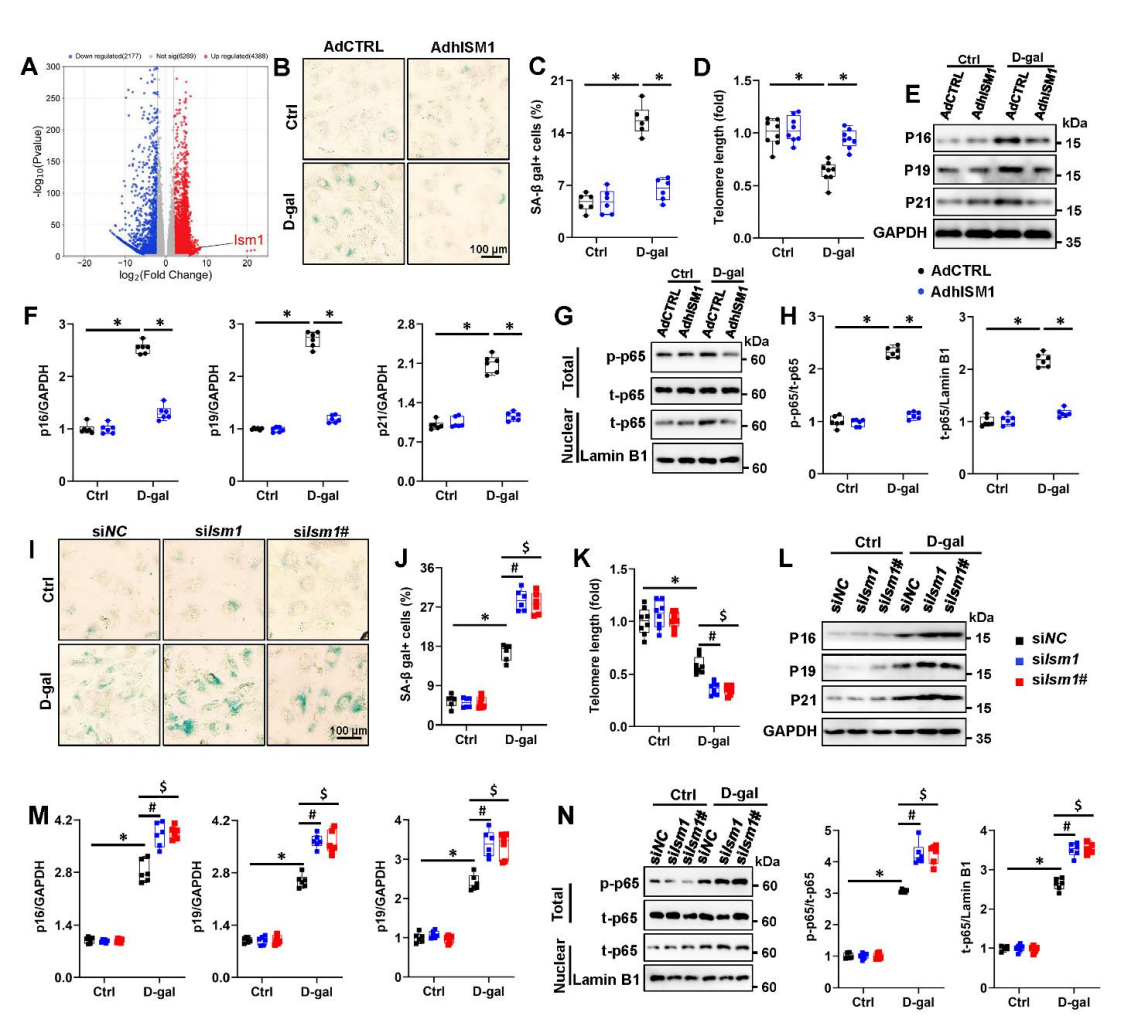
**ISM1 modulates D-gal-induced H9C2 cellar senescence. (A)** Volcano map of significantly different genes in the heart tissues from mice in 20M and 8M groups based on RNA-seq analysis. **(B-C)** Representative pictures of SA-β gal-stained cells and quantitative results (n=6). **(D)** Telomere length in cells relative to ctrl+Adctrl group (n=8). **(E-F)** Representative western blot images and statistical results (n=6). **(G-H)** Representative western blot images and statistical results (n=6). **(I-J)** Representative pictures of SA-β gal-stained cells and quantitative results (n=6). **(K)** Telomere length in cells relative to ctrl+Adctrl group (n=8). **(L-M)** Representative western blot images and statistical results (n=6). **(N)** Representative western blot images and statistical results (n=6). Comparisons between two groups were performed using an unpaired two-tailed Student′s *t*-test, whereas one-way analysis of variance followed by Tukey post hoc test was conducted for comparisons among three or more groups. Values represent the mean ± SEM. *P < 0.05 versus the matched group, # P < 0.05 si*Ism1* versus si*NC*, $ P < 0.05 si*Ism1*# versus si*NC*.

To identify key molecules that alleviate aging-related cardiac dysfunction, RNA sequencing was performed between aged mice (20M) hearts and young mice (8M) hearts. Compared with the 8M hearts, 4388 genes were significantly upregulated using a fold change of 4 and a p-value of 0.01 as cutoff values, and ISM1 was one of the 10 most significantly up regulated genes (Fig. 1A). To examine the involvement of ISM1 in aging-related dysfunction, we employed D-gal treatment, commonly used to establish an aging cell model, to induce cell senescence in H9C2 cells [30, 34]. As shown in Figure S1A, D-gal treatment increased ISM1level in H9C2 cells. Furthermore, SA-β gal staining and telomere length detection were applied to determine the role of ISM1 in D-gal-induced cellular senescence. As depicted in Figure 1B-D and Figure S1B, the numbers of SA-β gal-positive cells were significantly increased, while telomere length was decreased after D-gal treatment, which all were mitigated with ISM1 overexpression. Analogously, the protein levels of senescent markers including p16, p19, and p21 were reduced in ISM1-overexpressed cells upon D-gal treatment (Fig. 1E-F). Cardiac inflammaging is an important phenotype of cardiac aging. Emerging evidence has addressed a direct association between low-grade chronic inflammation and aging-related cardiac dysfunction, and our previous RNA-seq result indicated that overexpression ISM1 significantly inhibited inflammation related pathways (Supplementary Fig. 1C) [35]. So, we first determine the level of NF-κB that orchestrated the transcription of various inflammatory genes. As anticipated, ISM1 overexpression blocked the increase of phosphorylation and nuclear translocation of NF-κB p65 (Figure 1G-H). In addition, NLRP3 inflammasome, the sensor and regulator of inflammation was also decreased by ISM1 overexpression (Figure S1D). Briefly, it seems that ISM1 overexpression improved D-gal-induced H9C2 cellar senescence.

To further validate the involvement of ISM1 in cellar senescence, we silenced ISM1 with two independent si*RNAs* (si*Ism1* and si*Ism1#*) (Supplementary Fig. 1E-F). We found that D-gal-induced cellar senescence was exacerbated in ISM1-deficient H9C2 cells (Fig. 1I-M). On the other hand, ISM1 deficiency dramatically aggravated the phosphorylation and nuclear translocation of NF-κB p65 upon D-gal stimulation (Fig. 1N). Also, D-gal-induced increases in NLRP3 inflammasome were also enhanced by ISM1 silencing (Supplementary Fig. 1G). Collectively, our findings revealed that ISM1 assuaged D-gal-induced cellar senescence.

**Figure 2.**
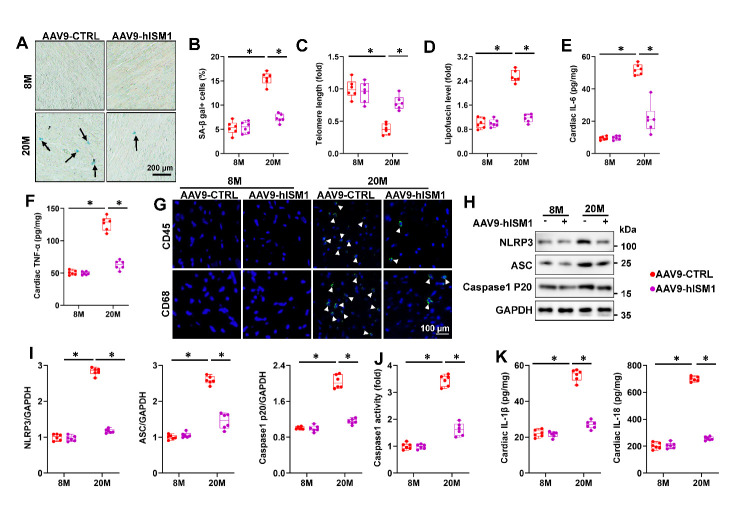
**ISM1 attenuates aging-related inflammatory response. (A-B)** Representative pictures of SA-β gal-stained heart sections and quantitative results (n=6). **(C)** telomere length in murine hearts relative to 8M+AAV9-CTRL group (n=6). **(D)** Cardiac lipofuscin content in murine hearts (n=6). **(E-F)** The myocardial IL-6 and TNF-α levels were determined by ELISA kits (n=6). **(G)** Representative image of CD45 and CD68 staining in heart sections (n=6). **(H-I)** Representative western blot images and statistical results (n=6). (J) The caspase1 activity in murine hearts (n=6). **(K)** The myocardial IL-1β and IL-18 levels were determined by ELISA kits (n=6). Comparisons between two groups were performed using an unpaired two-tailed Student′s *t*-test, whereas one-way analysis of variance followed by Tukey post hoc test was conducted for comparisons among three or more groups. Values represent the mean ± SEM. *P < 0.05 versus the matched group.

### ISM1 attenuates aging-related inflammatory response

To explore the potential role of ISM1 in aging-related cardiac dysfunction, we first determined ISM1 expression in the aging hearts. As depicted in Figure S2A-B, the protein and mRNA levels of ISM1 were increased in aging hearts. Next, we specifically overexpressed ISM1 in the myocardium with AAV9 vectors to further explore the role of ISM1 in aging-related cardiac dysfunction (Supplementary Fig. 2C). As shown in S2D-H, ISM1 overexpression did not affect the levels of serum TG, and TC, mean arterial pressure, FBG and heart rate. In addition, ISM1 overexpression decreased the number of SA-β gal-positive cells and preserved telomere length in aging hearts (Fig. 2A-C). Consistently, aging-related lipofuscin accumulation and senescent markers increase in aging hearts were suppressed by ISM1 overexpression (Fig. 2D and Supplementary Fig. 2I-J). Moreover, hISM1-infection decreased IL-6 and TNF-α levels in aging hearts (Fig. 2E-F and Supplementary Fig. 2K-L). Similarly, aging-induced infiltration of leukocytes was largely suppressed by ISM1 overexpression verified by immunohistochemistry staining (Fig. 2G). Aging-related upregulation of NLRP3, ASC, and cleaved caspase-1 p20 was prevented by ISM1 overexpression, accompanied by decreased cardiac caspase-1activity, IL-1β and IL-18 levels in aging hearts (Fig. 2H-K). Meanwhile, ISM1 overexpression dramatically reduced the phosphorylation and nuclear translocation of NF-κB p65 (Supplementary Fig. 2M). All of these results validated that ISM1 suppressed aging-associated inflammatory response.

### ISM1 attenuates aging-related cardiac dysfunction and remodeling

**Figure 3.**
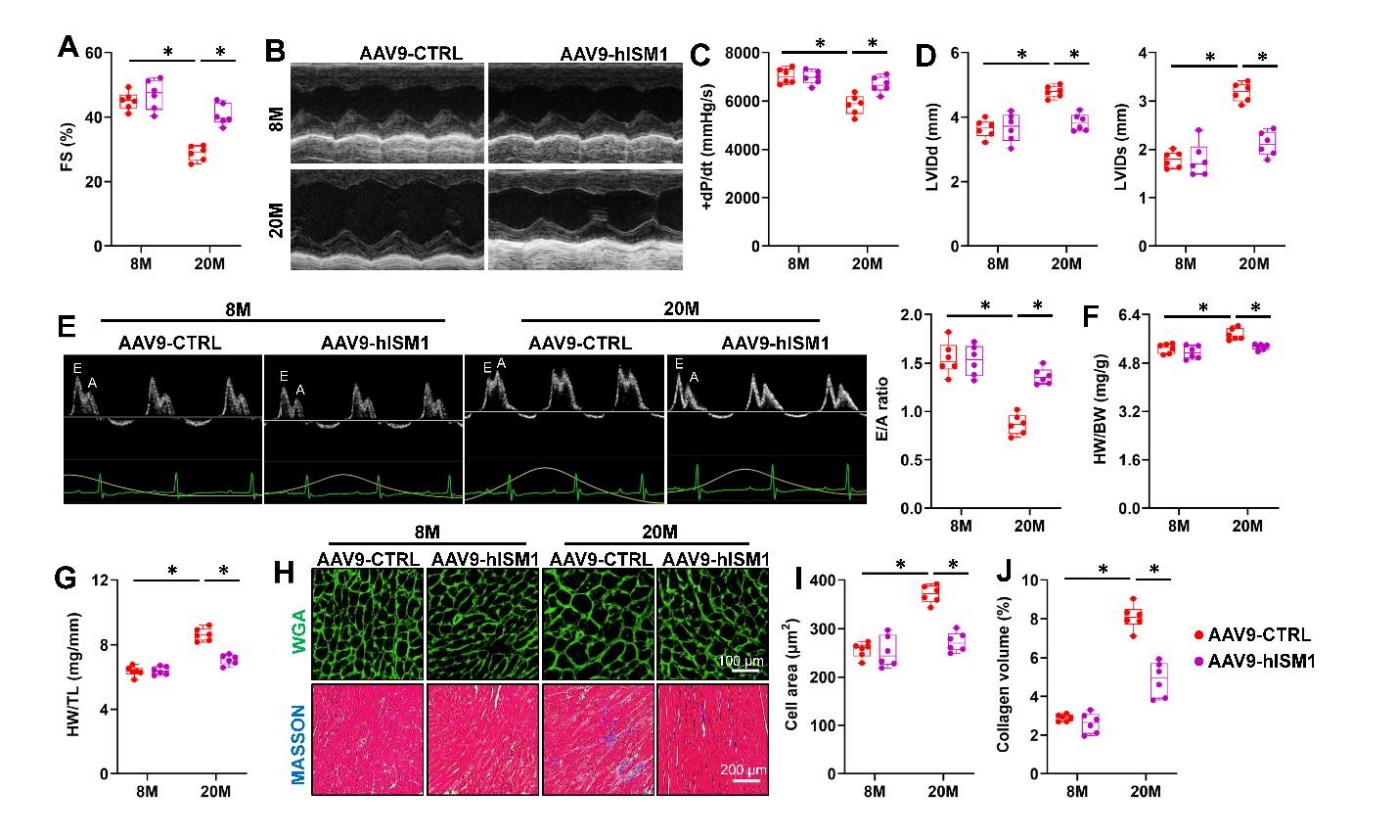
**ISM1 attenuates aging-related cardiac dysfunction and remodeling. (A)** Fractional shortening (FS) was determined by echocardiography (n=6). **(B)** Representative M-mode echocardiography was recorded. **(C)** The peak rates of isovolumic pressure development (+dP/dt) of mice (n=6). **(D)** left ventricular internal dimension at end-diastole (LVIDd) or end-systole (LVIDs) of mice were determined by echocardiography (n=6). **(E)** Representative image of the ratio of the early (E) to late (A) ventricular filling velocities and quantitative results (n=6). **(F)** Heart weight-to- Body weight (HW/BW) in mice (n=6). **(G)** Heart weight-to-Tibia length (HW/TL) in mice (n=6). **(H)** Representative image of Wheat germ agglutinin (WGA) and MASSON stainings in heart sections (n=6). **(I)** Quantification of the cardiomyocyte area with WGA staining (n=6). (J) Quantification of average collagen volume with MASSON staining (n=6). Comparisons between two groups were performed using an unpaired two-tailed Student′s *t*-test, whereas one-way analysis of variance followed by Tukey post hoc test was conducted for comparisons among three or more groups. Values represent the mean ± SEM. *P < 0.05 versus the matched group.

Aging myocardium undergoes progressive cardiac hypertrophy and interstitial fibrosis with diastolic and systolic dysfunction [36]. Aging-induced cardiac systolic dysfunction in mice was attenuated with ISM1 overexpression, as verified by the increased fractional shortening (FS) and the peak rates of isovolumic pressure development (+dP/dt) in left ventricles and decreased left ventricular internal dimension at end diastole (LVIDd) and end-systole (LVIDs) (Fig. 3A-D). Additionally, Aging-induced diastolic dysfunction was also improved by ISM1 overexpression verified by the increased ratio of the early (E) to late (A) ventricular filling velocities (Fig. 3E). Then we investigated whether ISM1 overexpression improved cardiac hypertrophy and interstitial fibrosis in aging hearts. As expected, AAV9-hISM1 infected mice showed decreased heart weight-to-body weight (HW/BW), heart weight-to-tibia length (HW/TL) and cardiomyocytes size (Fig. 3F-I). In addition, ISM1 overexpression blocked the activation of hypertrophic gene (Supplementary Fig. 3A). Accordingly, ISM1 overexpression markedly interrupted fibrotic remodeling in aging hearts, as verified by the decreased collagen volume and fibrotic markers levels (Fig. 3H and J and Supplementary Fig. 3B). To further determine the role of ISM1 in aging-related cardiac dysfunction, we treated mice with D-gal which accelerated mice aging displayed increased inflammation, pathological hypertrophy and collagen depositions [25]. As shown in Supplementary Figure 4A-D, D-gal-induced hearts exhibited significantly decreased telomere length and increased SA-β gal-positive cells, lipofuscin accumulation and senescent markers increase, which were improved by ISM1overexpression. Moreover, cardiac-specific ISM1 overexpression attenuated D-gal-induced cardiac inflammation and remodeling (Supplementary Fig. 4E-G). Together, these findings highlighted that ISM1 mitigated both natural and accelerated aging hearts dysfunction.

**Figure 4.**
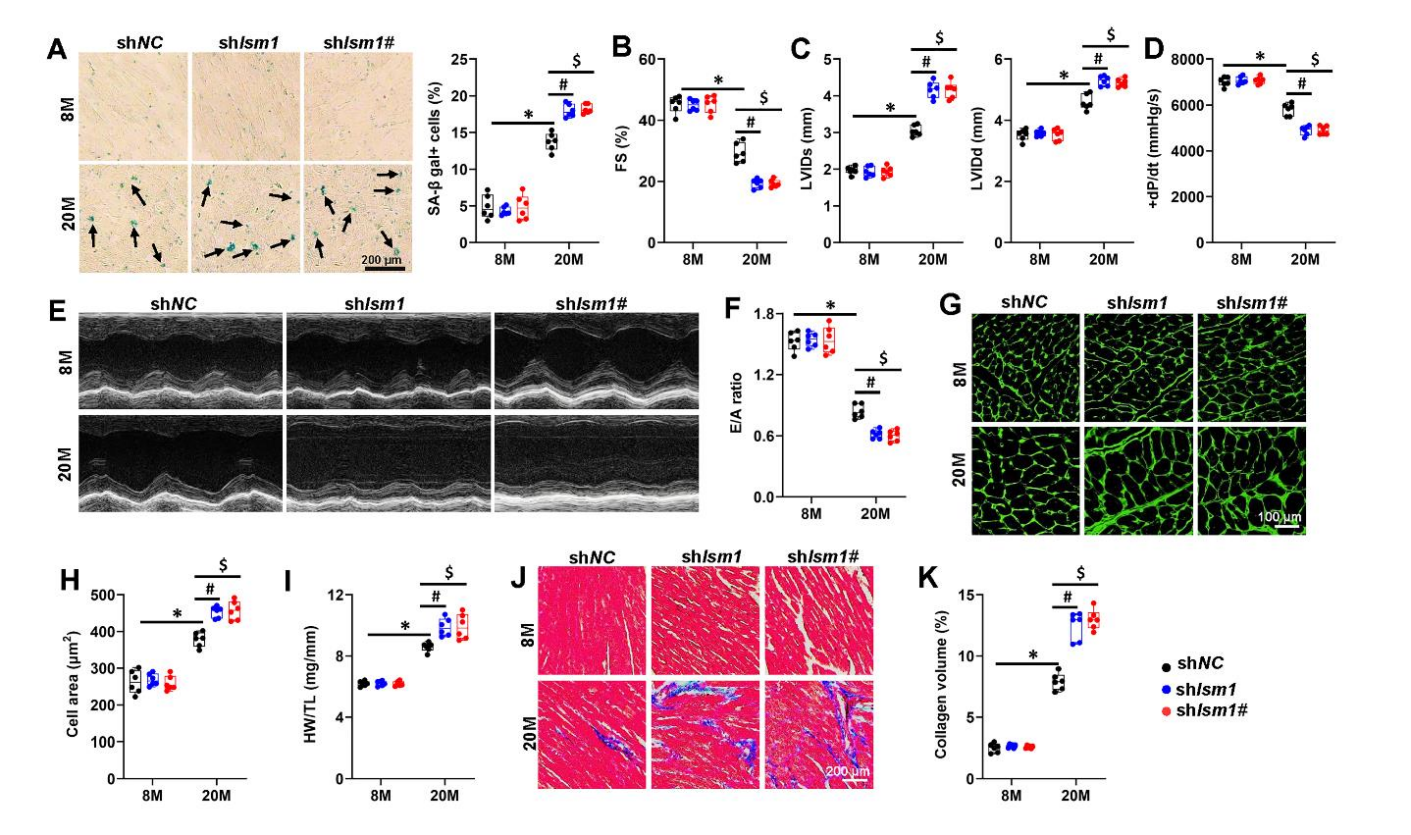
**ISM1 deficiency deteriorates aging-related cardiac dysfunction in mice. (A)** Representative pictures of SA-β gal-stained heart sections and quantitative results (n=6). **(B)** FS was determined by echocardiography (n=6). **(C)** LVIDd or end-systole LVIDs of mice were determined by echocardiography (n=6). **(D)** The peak rates of +dP/dt of mice (n=6). **(E)** Representative M-mode echocardiography was recorded. **(F)** The ratio of E/A (n=6). **(G-H)** Representative image of WGA and quantitative results (n=6). **(I)** HW/TL in mice (n=6). **(J-K)** Representative image of MASSON and quantitative results (n=6). Comparisons between two groups were performed using an unpaired two-tailed Student′s *t*-test, whereas one-way analysis of variance followed by Tukey post hoc test was conducted for comparisons among three or more groups. Values represent the mean ± SEM. *P < 0.05 versus the matched group, # P < 0.05 sh*Ism1* versus sh*NC*, $ P < 0.05 sh*Ism1*# versus sh*NC*.

### ISM1 deficiency deteriorates aging-related cardiac dysfunction in mice

To further investigate the role of ISM1, we specifically knocked down ISM1 in murine hearts using the AAV9 system with two independent shRNAs against mouse *Ism1* (sh*Ism1* and sh*Ism1*#) (Supplementary Fig. 5A-B). In addition, ISM1 silencing dramatically facilitated cellar senescence, as identified by increased SA-β gal-positive cells and decreased telomere length (Fig. 4A and Supplementary Fig. 5C). Aging-related lipofuscin accumulation and senescent markers increase in aging hearts were further promoted by ISM1 deficiency (Supplementary Fig. 5D-E). Accordingly, cardiac-specific ISM1 silencing exacerbated aging-induced cardiac IL-6 and TNF-α increase (Supplementary Fig. 6A). Aging-related upregulation of NLRP3, ASC, and cleaved caspase-1 p20 was aggravated by ISM1 silencing, accompanied by increased cardiac caspase-1activity, IL-1β and IL-18 levels in aging hearts (Supplementary Fig. 6 B-E). Meanwhile, ISM1-silenced aging mice exhibited significantly increased NF-κB p65 phosphorylation and nuclear translocation (Supplementary Fig. 6F). Consistently, aging-induced cardiac systolic dysfunction and diastolic dysfunction were exacerbated in ISM1-silenced hearts (Fig. 4B-F). Furthermore, disruption of ISM1 accelerated cardiac hypertrophy and fibrotic remodeling in aging hearts (Fig. 4G-K). Similarly, ISM1silencing further deteriorated D-gal-induced mice aging (Supplementary Fig. 7A-D). Accordingly, ISM1-silenced mice exhibited significantly increased inflammation and remodeling (Supplementary Fig. 7E-I). Collectively, our findings revealed that cardiac-specific ISM1 knockdown exacerbated both natural and accelerated aging hearts dysfunction.

### ISM1 protects against aging-related cardiac dysfunction by promoting glycolysis and enhancing HBP flux

**Figure 5.**
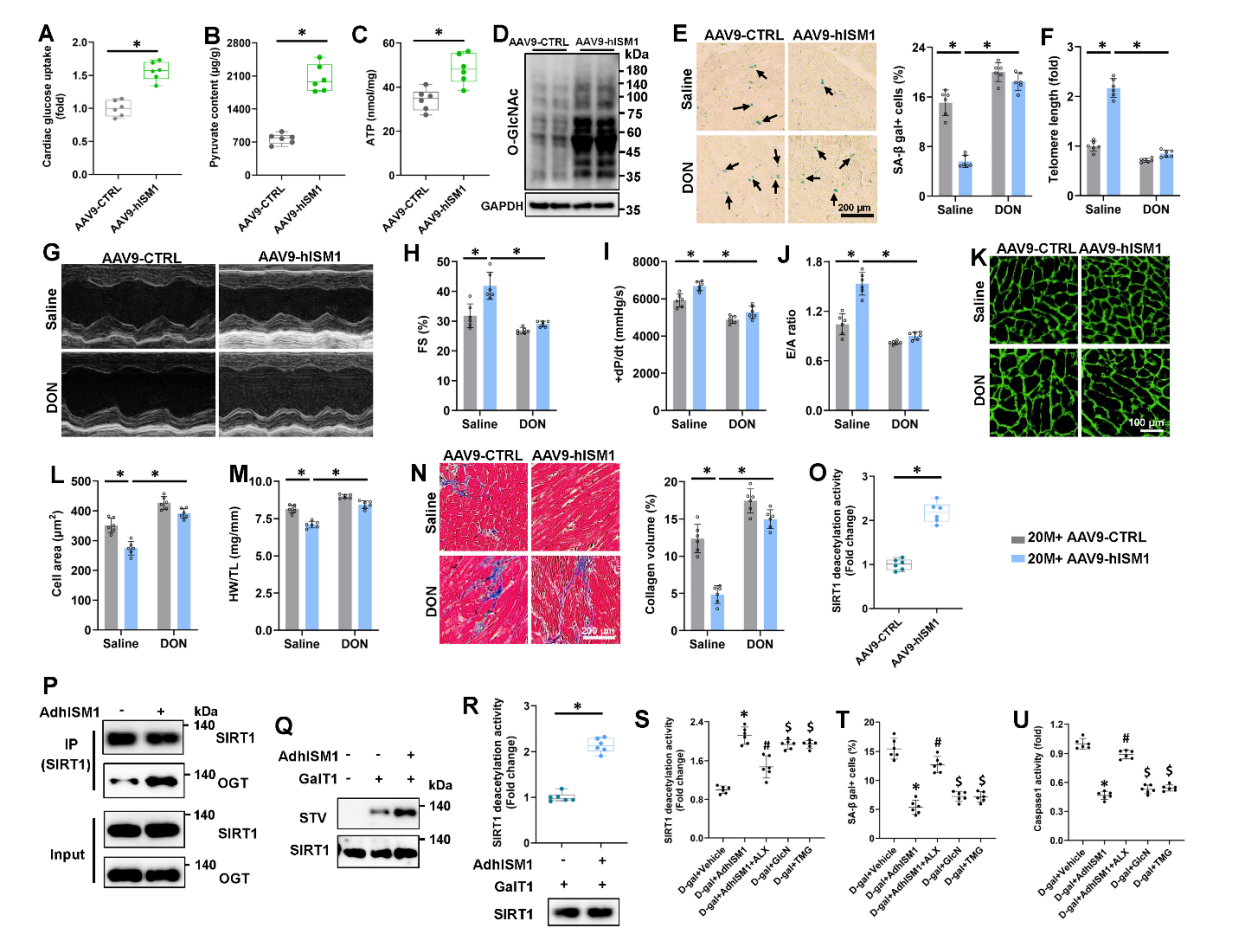
**ISM1 protects against aging-related cardiac dysfunction by promoting glycolysis and enhancing HBP flux. (A)** Glucose uptake in hearts (n=6). **(B)** Pyruvate content in hearts (n=6). **(C)** ATP level in hearts (n=6). **(D)** Representative western blot images (n=6). **(E)** Representative pictures of SA-β gal-stained heart sections and quantitative results (n=6). **(F)** Telomere length in murine hearts relative to saline+20M+AAV9-CTRL group (n=6). **(G)** Representative M-mode echocardiography was recorded. **(H)** FS was determined by echocardiography (n=6). **(I)** The peak rates of +dP/dt of mice (n=6). **(J)** The ratio of E/A (n=6). **(K-L)** Representative image of WGA and quantitative results (n=6). **(M)** HW/TL in mice (n=6). **(N)** Representative image of MASSON and quantitative results (n=6). **(O)** SIRT1 deacetylase activity in hearts (n=6). **(P)** H9C2 cells were immunoprecipitated with antiSIRT1, and the precipitates were analyzed by western blotting. Q O-GlcNAcylation of SIRT1 was detected by chemoenzymatic labeling and IB analysis. R SIRT1 deacetylase activity in H9C2 cells (n=6). (S) SIRT1 deacetylase activity in H9C2 cells (n=6). **(T)** Quantitative result of SA-β gal-stained cells (n=6). **(U)** Caspase1 activity in H9C2 cells (n=6). Comparisons between two groups were performed using an unpaired two-tailed Student′s t-test, whereas one-way analysis of variance followed by Tukey post hoc test was conducted for comparisons among three or more groups. Values represent the mean ± SEM. *P < 0.05 versus the matched group, #P<0.05 versus “D-gal +AdhISM1” group, $P<0.05 versus “D-gal +AdhISM1+ALX” group.

Lipid catabolism is a crucial point in the development of heart aging development and the mismatch between lipid uptake and oxidation drives cardio-lipotoxicity and partly accounts for insulin resistance during aging. Herein, mRNA levels of genes related to lipid metabolism, CD36 protein levels and rate-limiting enzyme activities were detected in cardiomyocytes. However, ISM1 over-expression did not affect CD36 level and major rate-limiting enzyme activity in both aging mice (Supplementary Fig. 8). To investigate whether the beneficial effects of ISM1 on aging-related cardiac dysfunction are related to its close association with glucose metabolism, we first examined the glucose uptake in the aging hearts after ISM1 overexpression. As shown in Figure 5A, ISM1 overexpression significantly increased glucose uptake in aging hearts. To further explore whether ISM1 protected against aging-related cardiac dysfunction by increasing glucose uptake, we first investigated whether the increased glucose uptake was used for glycogen storage. Fallaciously, enhanced glucose uptake was not stored, as identified by unchanged glycogen level (Supplementary Fig. 9A). Then, we focused on three primary catabolism including glycolysis, pentose phosphate pathway and oxidative phosphorylation. As shown in Figure 5B and Supplementary Figure 9B-C, pyruvate increased after ISM1 overexpression in aging hearts while NADPH level and citrate synthase activity (a marker of mitochondrial capacity) exhibited no appreciable differences. Accordingly, ISM1 overexpression enhanced ATP production via enhancing glycolysis flux (Fig. 5C). Increased glucose uptake in the heart is associated not only with an increase in flux through glycolysis but also with increased flux via other pathways include the HBP that utilize glycolytic intermediates [13]. N-acetylglucosamine (UDP-GlcNAc) generated from the HBP plays key roles in many of the major diseases associated with aging via O-linked GlcNAc (O-GlcNAc) protein modifications [37]. Besides, Chatham and colleagues as well as we have shown that increased HBP flux and increased protein O-glycosylation may increase the resistance of hearts to hypertrophy and remodeling [38, 39]. As anticipated, aging mice infected with AAV9-hISM1 shown significantly elevated O-GlcNAcylation protein level (Fig. 5D). In addition, ISM1 overexpression did not the activity of rate-limiting enzyme in glycolysis and HBP (Fig. S9D-E). To confirm whether enhanced HBP-O-GlcNAc signaling was involved in the protection of ISM1 in aging mice, we administrated DON to inhibit HBP flux in AAV9-hISM1 infected mice. DON treatment partly blunted the alleviation role of ISM1 in aging mice, as evidenced by the increased levels of SA-β gal positive cells and senescent markers levels and decreased telomere length (Fig. 5E-F and Supplementary Fig. 9F-G). Concomitantly, the inhibitory effects of ISM1 on aging-induced cardiac dysfunction and remodeling were also retarded by DON administration (Fig. 5G-N). Besides, ISM1 overexpression failed to fully mitigate inflammation in aging hearts after DON administration (Supplementary Fig. 9H-I). In summary, these data indicated that the protection of ISM1 in aging-related cardiac dysfunction may be associated with enhanced glycolysis and HBP flux.

### HBP flux modified SIRT1 with O-GlcNAc at Ser 549

AMPKα is an energy sensor protein kinase activated in response to reduction of intracellular ATP levels and the activity of AMPKα is positively regulated by O-GlcNAcylation [40, 41]. Besides, our recent study proved that fibronectin type III domain-containing 5 improved aging-related cardiac dysfunction by activating AMPKα [27]. Fallaciously, cardiac-specific ISM1 overexpression could not activate AMPKα, as determined by the unchanged phosphorylation of AMPKα and the downstream acetyl CoA carboxylase ACC phosphorylation in aging murine hearts (Supplementary Fig. 10A-B). SIRT1 is the other nutrient deprivation signal that sensitive to glucose deprivation and cellular stress, and activation of SIRT1 has been shown to extend the lifespan and improve aging-related diseases in a variety of organisms [42-44]. As expected, ISM1 overexpression significantly elevated SIRT1 deacetylase activity in aging hearts (Fig. 5O). We first determined whether ISM1 overexpression increases the SIRT1 level. Disappointingly, no alteration of SIRT1 levels in aging hearts was observed after ISM1 overexpression (Supplementary Fig. 10C-E). SIRT1 is a well-known NAD+ dependent deacetylase and its activation not only involves an upregulation of protein abundance but also depends on the increase of NAD+ concentrations [45]. Amusingly, ISM1 overexpression did not alter NAD+ level in aging hearts (Supplementary Fig. 10F). Besides, the cAMP acts as an important second messenger and is proved to activate SIRT1 via NAD+ independent manners [46]. Thus, we detect the cAMP level but identified that ISM1 did not increase cAMP abundance in aging hearts (Supplementary Fig. 10G). Yu and coworkers demonstrated that O-GlcNAcylation of SIRT1 enhances its deacetylase activity and promotes cytoprotection under stress [47]. In the above study, we determined increases of O-GlcNAcylation protein level by ISM1, which prompted us to address the possibility that SIRT1 is modified with O-GlcNAc after ISM1 overexpression. To determine the hypothesis, we first investigated whether OGT could bind to SIRT1 to evaluate the possible links between O-GlcNAcylation and SIRT1after ISM1 overexpression, as OGT is the only known cytonuclear enzyme for intracellular protein O-GlcNAcylation. As shown in Figure 5P, endogenous OGT was co-immunoprecipitated with the SIRT1 specific antibody, and ISM1 overexpression increased the precipitation of OGT in H9C2 cells. Additionally, the SIRT1 proteins were immunoprecipitated and detected by probing with streptavidin-HRP (STV-HRP). The results indicated that SIRT1 in H9C2 cells could be labeled by GlcNAz, and that the modification of SIRT1 was enhanced by overexpression of ISM1 (Fig. 5Q). Furthermore, the deacetylase activities of immunopurified SIRT1 were measured using an in vitro fluorometric assay with a p53 peptide acetylated at K382 as the substrate. The results showed that ISM1 overexpression enhanced the deacetylase activity of SIRT1 (Fig. 5R). To further validate ISM1 ameliorated cardiomyocyte injury through stimulating SIRT1 O-GlcNAcylation in vitro, D-gal-induced H9C2 cells with AdhISM1 infection were treated with alloxan monohydrate (ALX), an OGT inhibitor, as shown in Supplementary Figure 5, the increased SIRT1 activity was counteracted by ALX. In contrast, the deleterious effect of aging on SIRT1 activity was improved by supplementation of glucosamine hydrochloride (GlcN) or by thiamet G (TMG, a OGA inhibitor). Accordingly, OGA inhibition or GlcN supplementation showed advantageous effects on D-gal-induced cells, similar to ISM1 overexpression, while ALX played a deleterious role (Fig. 5T-U). Collectively, these findings implied that the SIRT1 O-GlcNAcylation was involved in ISM1-mediated cardioprotection *in vitro*.

**Figure 6.**
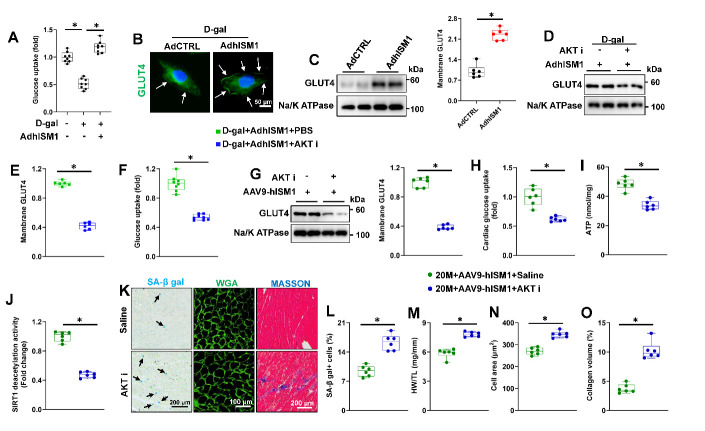
**ISM1 promotes glucose uptake in cardiomyocytes via translocating GLUT4 to the cell surface. (A)** Glucose uptake in H9C2 cells (n=8). **(B)** Representative image of GLUT4 staining in H9C2 (n=6). **(C)** Representative western blot images and statistical results (n=6). **(D-E)** Representative western blot images and statistical results (n=6). **(F)** Glucose uptake level in H9C2 cells (n=8). **(G)** Representative western blot images and statistical results (n=6). **(H)** Glucose uptake level in hearts (n=6). **(I)** ATP level in hearts (n=6). **(J)** SIRT1 deacetylase activity in hearts (n=6). **(K)** Representative image of SA-β gal, WGA and MASSON (n=6). **(L)** The quantitative results of SA-β gal staining (n=6). (M) HW/TL in mice (n=6). **(N)** The quantitative results of WGA (n=6). **(O)** The quantitative results of MASSON (n=6). Comparisons between two groups were performed using an unpaired two-tailed Student′s *t*-test, whereas one-way analysis of variance followed by Tukey post hoc test was conducted for comparisons among three or more groups. Values represent the mean ± SEM. *P < 0.05 versus the matched group.

To further confirm the implication of the SIRT1 O-GlcNAcylation in ISM1-mediated cardioprotection in vivo, we first investigated the SIRT1 activity. As shown in Figure S11A, SIRT1 activity was significantly increased after ISM1 overexpression, which was blocked by DON administration. We also used cardiac-restricted *Sirt1* knockout (cKO) mice to gain evidence that SIRT1 activation was responsible for the cardioprotective effects of ISM1 in aging mice (Supplementary Fig. 11B) [18]. As depicted in Supplementary Figure 11C-F, ISM1 failed to decrease the senescent markers levels and SA-β gal-positive cells, and also failed to increase telomere length. SIRT1 deficiency completely abolished ISM1 overexpression-mediated suppression of inflammation (Fig. S11G-H). Furthermore, the improved systolic and diastolic function in aging hearts with ISM1 overexpression was evidently negated by SIRT1 deficiency (Fig. S12A-B). ISM1 lost its protective effects against cardiac hypertrophy and fibrosis after SIRT1 deficiency (Supplementary Fig. 12C-G). Collectively, these findings implied that SIRT1 modified with O-GlcNAc was involved in ISM1-mediated cardioprotection against aging hearts. Yu and coworkers identified that SIRT1 is dynamically modified with O-GlcNAc at Ser 549 in its carboxy-terminal region, which directly increases its deacetylase activity [47]. To validate whether S549 is a major site of O-GlcNAcylation, we overexpressed SIRT1 with an alanine substitution at S549 site (SIRT1^S549A^) or wtSIRT1 in SIRT1 silenced H9C2 cells. As depicted in Figure S13A-B, SIRT1^S549A^ blocked ISM1 overexpression-mediated the increased deacetylase activity in D-gal-stimulated H9C2 cells. Accordingly, the decreased SA-β gal-positive cells in ISM1-overexpressed H9C2 were dramatically blunted in SIRT1^S549A^ mice (Supplementary Fig. 13C). Meanwhile, ISM1 lost its inhibitory effects on p16, p19, and p21 expressions in SIRT1^S549A^ D-gal-stimulated cells (Supplementary Fig. 13D-E). The inhibitory effect of ISM1 on inflammation was retarded by SIRT1^S549A^ (Supplementary Fig. 13F). These findings suggested that O-GlcNAcylation of SIRT1 at S549 was a molecular switch that mediated the protected role of ISM1 in aging hearts.

### ISM1 promotes glucose uptake in cardiomyocytes via translocating GLUT4 to the cell surface

As shown in Figure 6A, ISM1 overexpression also increased glucose uptake in vitro. Svensson et al. determined that ISM1 increased adipocyte glucose uptake by translocating GLUT4 to the cell surface via PI3K-AKT pathway [21]. As shown in Figure 6B-C and Supplementary Figure 14A, ISM1 overexpression induced higher level of GLUT4 at cell surface in D-gal stimulated H9C2. Notably, AKT i treatment significantly restrained the GLUT4 translocation and glucose uptake in D-gal-induced cells (Fig. 6D-F). AKT i treatment also blunted the alleviation of cellular senescence by ISM1 overexpression, as evidenced by the increased SA-β gal-positive cells (Supplementary Fig. 14B-C). Accordingly, ISM1 silence significantly blocked the activation of AKT, decreasing the membrane translocation of GLUT4, and eventually reduced glucose uptake in H9C2 cells (Supplementary Fig. 14D-I). Meanwhile, ISM1 overexpression increased GLUT4 translocated to the cell surface in aging mice (Supplementary Fig. 15A). AKT i treatment blocked the translocation of GLUT4 (Fig. 6G). More importantly, the increased glucose uptake, ATP production and SIRT1 deacetylase activity were restrained with AKT i treatment (Fig. 6H-J). Also, AKT i administration blunted the alleviation of cellular senescence by ISM1 overexpression in aging hearts (Fig. 8K-L and Supplementary Fig. 15B-D). Concomitantly, the inhibitory effects of ISM1 on inflammation were also abrogated by AKT i administration Supplementary (Fig. 15E-F). Correspondingly, ISM1 overexpression failed to restore cardiac dysfunction after AKT i administration (Supplementary Fig. 15G-I). Besides, ISM1 lost its protective effects against cardiac hypertrophy and remodeling after AKT i treatment (Fig. 6K-O and Supplementary Fig. 15J).

Taken together, our data identify that ISM1 facilitated the membrane translocation of GLUT4 mediated by AKT to increase the glucose uptake and ultimately mitigated aging-related cardiac dysfunction.

### RISM1 infusion mitigates aging-related cardiac dysfunction

Given the cardioprotective role of ISM1 in aging mice, we sought to determine whether rISM1 infusion for 2 months would attenuate aging-induced cardiac dysfunction. As shown in Supplementary Figure 16A-B, rISM1 infusion significantly reduced the numbers of SA-β-gal positive cells and decreased the protein levels of senescent markers in aging hearts. Additionally, aging-induced cardiac systolic and diastolic dysfunction were improved by rISM1 treatment (Supplementary Fig. 16C-E). RISM1 infusion also attenuated aging-induced cardiac remodeling, as evidenced by the improvement of cardiac hypertrophy and fibrosis (Supplementary Fig. 16F-H). Consistent with the phenotypic alterations in vivo, rISM1 treatment dramatically decreased the SA-β-gal-positive cells and decreased the protein levels of senescent markers induced by D-gai in vitro (Supplementary Fig. 17A-B). To examine the clinical impact of ISM1 on aging-related cardiac dysfunction, we evaluated the serum level of ISM1 in people. As shown in Supplementary Figure 17C, the elderly people (over 60 years old) exhibited a significant increase of serum ISM1level compared with the young people (<60 years old). In addition, the increased serum ISM1 in the elderly predicted a predicted a decrease of serum NT-proBNP and an increase of LVEF (Fig. S17D-E). Besides, we divided the elderly into low ISM1 and high ISM1 groups using the median of serum ISM1 as the threshold. Compared with the low ISM1 group, the elderly with higher serum ISM1 levels exhibited lower NT-proBNP levels and high LVEF (Supplementary Fig. 17F). All these data revealed that ISM1 might be a novel potential therapeutic target for preventing age-related cardiac dysfunction.

## DISCUSSION

In this study, we verify that ISM1may be a promise therapeutic target for age-related cardiac disease. Cardiac-specific overexpression of ISM1 significantly suppresses cardiac inflammation and alleviate cellular senescence and cardiac dysfunction in aging mice, while cardiac specific silence ISM1 deteriorates cellular senescence and cardiac dysfunction in aging mice. Mechanistically, we prove that ISM1 enhances glycolysis and HBP flux through increased glucose uptake via translocating GLUT4 to the cell surface. More importantly, we utilize *Sirt1* cKO mice and AAV9- SIRT1^S549A^ and determine that HBP flux active SIRT1 through O-GlcNAcylation of SIRT1 at S549, thus increase SIRT1 deacetylase activity. Regrettably, we did not compare the dominance of enhanced glycolysis and increased SIRT1 activity in ISM1-mediated aging-related cardiac protection. Hence, our present study identifies a novel potential therapeutic target for preventing age-related cardiac dysfunction.

Aging is a natural and complex biological process that is associated with widespread functional declines in numerous physiological processes, terminally affecting multiple organs and tissues [48]. Currently, the prevalence of aging-induced cardiovascular disease gradually increases, thus, aging has become one of the main risk factors for cardiovascular diseases, which mainly exhibited with structure and function of the heart deteriorates with age [49-51]. Therefore, it’s very important to explore effective therapeutic target to alleviate aging-induced cardiac dysfunction. Previous study identified that anaerobic glycolysis gradually dominates the energy source in the aging heart instead of lipid catabolism and glucose oxidation, that may because of impaired cardiac mitochondria ability including mitochondrial content decreases, interfibrillar mitochondria defective, cytochrome oxidase enzyme activity decrease and mitochondrial biogenesis impairment in aging hearts [52-55]. It has been reported that the ability that glucose transporters (GLUTs) to transfer glucose is decreased during aging, and the major isoform switches from GLUT1 to GLUT4 in heart with ageing [5, 56]. A major rate-governing kinetic step in overall myocellular glucose utilization is cardiac glucose uptake under insulin resistant condition [57]. Also, Boer et al. also determined that increased glucose utilization increases O-GlcNAc signaling and figure vigorous role in cardiac hypertrophy and remodeling [58]. Tian et al. shown that increased insulin-independent glucose transport and utilization in mouse hearts subjected to pressure overload blunted the progression to failure [10]. In summary, the present study provides strong evidence to support the potential benefit of increasing glucose utilization in the aging heart. Our results confirm the speculation from Abel that in addition to ATP production through glycolysis, there may be additional mechanisms exist for the potential benefits of myocardial glucose utilization during aging [13]. Previous studies identified that cardiac myocytes exposed to high levels of glucose have demonstrated contractile dysfunction and cell death that are dependent on the increases in intracellular glucose, which suggests that increased glucose entry causes glucotoxicity [59, 60]. Nevertheless, studies identified that despite 2-fold higher than maximum insulin stimulated glucose uptake in WT, the cardiac morphology and function are not different from normal WT mice during the 2-year, and life-long increases in glucose uptake result in a favorable metabolic phenotype that affords protections against aging-associated increase of susceptibility to ischemic injury [9, 10].

In conclusion, we verified that ISM1 improves aging-related cardiac dysfunction by promoting glycolysis and enhancing SIRT1 deacetylase activity, and it might be a promising therapeutic target to decrease the mortality and increase the quality of live in the elderly. While we have identified ISM1 as a potential therapeutic target for age-related cardiac dysfunction, further validation is required to translate the research findings into clinical applications. In the future, we plan to conduct additional clinical studies to evaluate the safety and efficacy of targeting ISM1 in the human population, as well as assess the dosage, long-term effects, and potential side effects of recombinant ISM1 in humans.

## Supplementary Materials

The Supplementary data can be found online at: www.aginganddisease.org/EN/10.14336/AD.2024.0113.

## Data Availability

All data that support the findings of this study are available from the corresponding author upon reasonable request.
