# Supplementary material for: Isthmin-1 Improves Aging-Related Cardiac Dysfunction in Mice through Enhancing Glycolysis and SIRT1 Deacetylase Activity
Source: Aging Dis. 2024 Jan 25;15(6):2682–96. doi: 10.14336/AD.2024.0113 (PMC11567257; doi:10.14336/AD.2024.0113)
Supplement: Supplementary file 1 — The Supplementary data can be found online at: www.aginganddisease.org/EN/10.14336/AD.2024.0113. [file AD-15-6-2682-s.pdf]

SUPPLEMENTARY DATA

Isthmin-1 Improves Aging-Related Cardiac Dysfunction in Mice through Enhancing Glycolysis and SIRT1 Deacetylase Activity

Min Hu, Xin Zhang, Yi-Peng Gao, Yu-Xin Hu, Teng Teng, Sha-Sha Wang, Qi-Zhu Tang

© 2024. Hu M et al. Published online at http://www.aginganddisease.org/EN/10.14336/AD.2024.0113

SUPPLEMENTARY DATA

Supplementary Table 1. The antibody used in the article.

REAGENT	SOURCE	IDENTIFIER
Anti-ISM1 antibody	Abcam	ab275610
O-GlcNAc (ctd110.6)	Cell Signaling Technology	#3724
p-P65	Cell Signaling Technology	#3033
t-P65	Cell Signaling Technology	#8242
GAPDH Rabbit mAb	Cell Signaling Technology	#2118
GLUT4	Cell Signaling Technology	#2213
AKT	Cell Signaling Technology	#4691
p-AKT	Cell Signaling Technology	#4060
p16	Santa Cruz Biotechnology	sc-1661
p19	Santa Cruz Biotechnology	sc-32748
p21	Santa Cruz Biotechnology	sc-6246
Lamin B1 Rabbit mAb	Cell Signaling Technology	#17416
NLRP3	Novus Biologicals	IMG-6668A
ASC	Santa Cruz Biotechnology	sc-514414
Caspase1 p20	Proteintech	22915-1-AP
Hexokinase II (C64G5)	Cell Signaling Technology	#2867
CD36	Proteintech	18836-1-AP
SIRT1	Cell Signaling Technology	#8469
P-AMPK α	Cell Signaling Technology	#2537
T-AMPK α	Cell Signaling Technology	#2603
P-ACC	Cell Signaling Technology	#3662
T-ACC	Cell Signaling Technology	#11818
GFPT1	Proteintech	14132-1-AP

Supplementary Table 2. The primers used in quantitative real-time PCR.

**Species**	**Gene**	**Forward primer**	**Reverse primer**
Mice	*Ism1*	GATGGCCCTGACTCCGAAG	GGTCCCCACTATTGTCCTGG
Mice	*Anp*	ACCTGCTAGACCACCTGGAG	CCTTGGCTGTTATCTTCGGTACCGG
Mice	*α-Mhc*	GGATGCCCTGCTGGTTA	CGCCCAAACTCCTCCTT
Mice	*β-Mhc*	CCGAGTCCCAGGTCAACAA	CTTCACGGGCACCCTTGGA
Mice	*Col1α1*	AGGCTTCAGTGGT T TGGATG	CACCAACAGCACCATCGTTA
Mice	*Col3α1*	CCCAACCCAGAGATCCCATT	GAAGCACAGGAGCAGGTGTAGA
Mice	*Il-6*	AGTTGCCTTCTTGGGACTGA	TCCACGATTTCCCAGAGAAC
Mice	*Tnf-α*	GCAAAGGGAGAGTGGTCA	CTGGCTCTGTGAGGAAGG
Mice	*Gapdh*	ACTCCACTCACGGCAAATTC	TCTCCATGGTGGTGACGACA
Rat	*GAPDH*	GACATGCCGCCTGGAGAAAC	AGCCCAGGATGCCCTTTAGT
Rat	*ISM1*	CGGTGAGGGTGATTGGA	ATTCCTGGGCAGTTTGGA
Rat	*Anp*	CGGTACCGAAGATAACAGCCA	TCACCACCTCTCAGTGGCAA
Rat	*α-Mhc*	CAGAAAATGCACCATGAGGA	TCAAGCATTCATATTTATTGTGGC
Rat	*β-Mhc*	GCTCCTAAGTAATCTGTTTG	AAGTGAGGGTGCGTGGAGCG

© 2024. Hu M et al. Published online at http://www.aginganddisease.org/EN/10.14336/AD.2024.0113

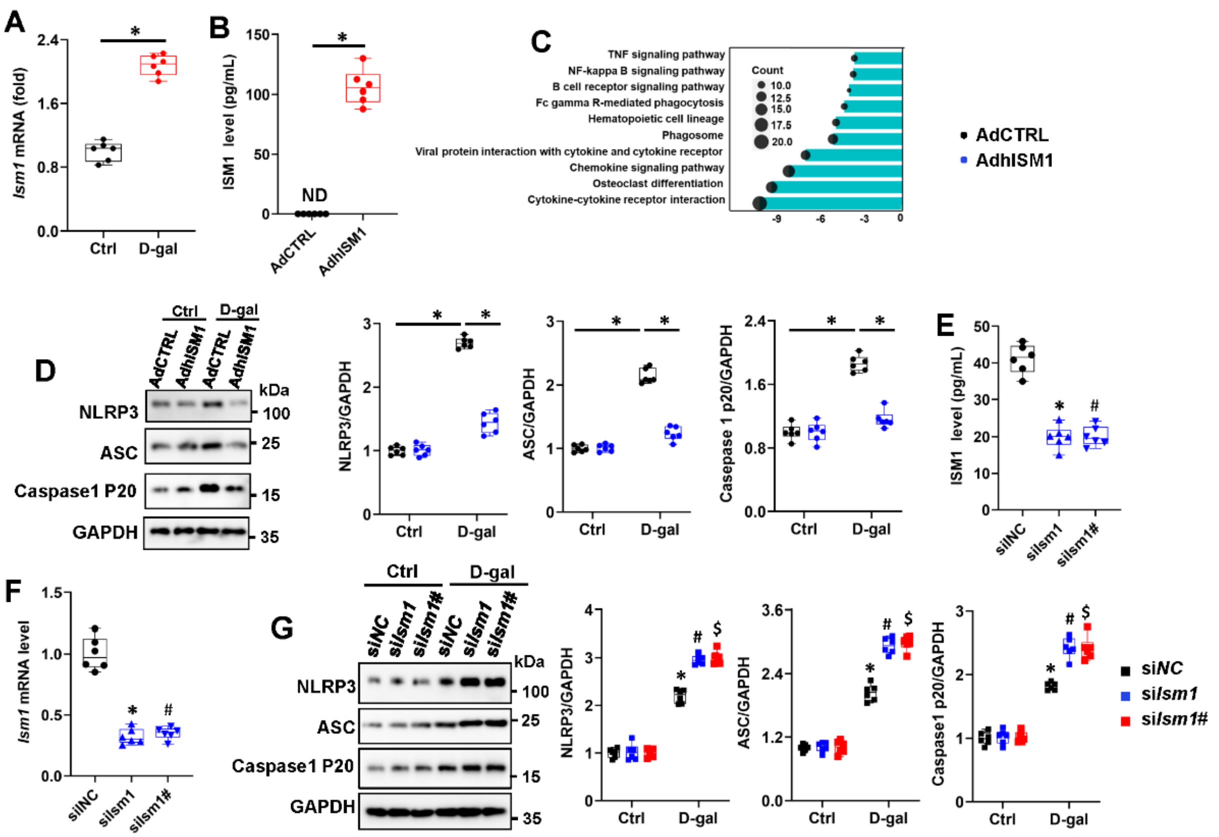

Supplementary Figure 1. ISM1 modulates D-gal-induced cellular senescence in H9C2. **A** Relative *Ism1* mRNA level in cells (n=6). **B** The ISM1 level was determined by ELISA kits (n=6). **C** KEGG analysis of RNA-seq. **D-E** Representative western blot images and statistical results (n=6). **F** The ISM1 level was determined by ELISA kits (n=6). **G** Relative *Ism1* mRNA level in cells (n=6). **H** Representative western blot images and statistical results (n=6). Comparisons between two groups were performed using an unpaired two-tailed Student's *t*-test, whereas one-way analysis of variance followed by Tukey post hoc test was conducted for comparisons among three or more groups. Values represent the mean ± SEM. *P < 0.05 versus the matched group, # P < 0.05 si*Ism1* versus si*NC*, $ P < 0.05 si*Ism1*# versus si*NC*.

SUPPLEMENTARY DATA

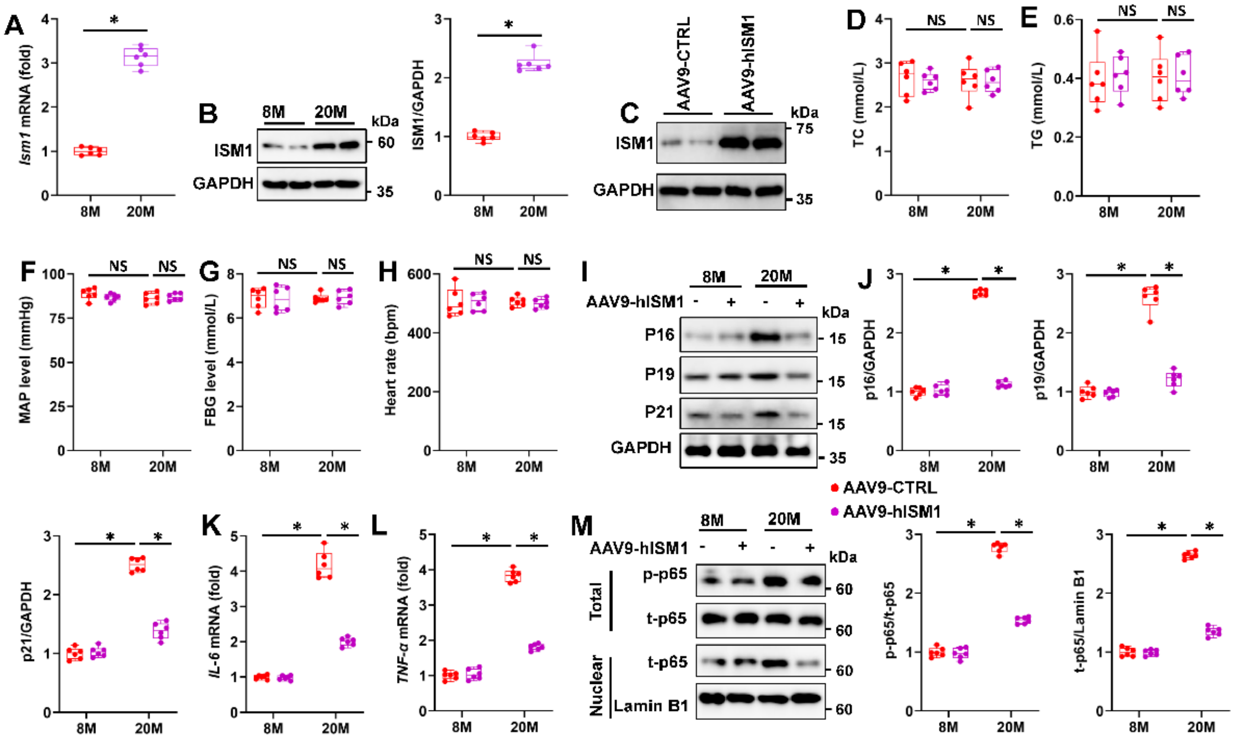

Supplementary Figure 2. ISM1 attenuates aging-related inflammatory response. A Relative *Ism1* mRNA level in hearts (n=6). **B** Representative western blot images and statistical results (n=6). **C** Representative western blot images (n=6). **D-E** The serum total cholesterol (TC) and triglyceride (TG) levels among groups (n=6). **F** 6-M-old young and 18-M-old aging mice were injected with AAV9-hISM1 for 8 weeks to overexpress hISM1 or AAV9-CTRL as a control, and then mean arterial pressure (MAP) was determined in mice among groups (n=6). **G** Fasting blood glucose (FBG) in mice among groups (n=6). **H** Heart rate among groups (n=6). **I-J** Representative western blot images and statistical results (n=6). **K-L** the mRNA levels of myocardial interleukin-6 (IL-6) and tumor necrosis factor-α (TNF-α) (n=6). **M** Representative western blot images and statistical results (n=6). Comparisons between two groups were performed using an unpaired two-tailed Student's *t*-test, whereas one-way analysis of variance followed by Tukey post hoc test was conducted for comparisons among three or more groups. Values represent the mean ± SEM. *P < 0.05 versus the matched group.

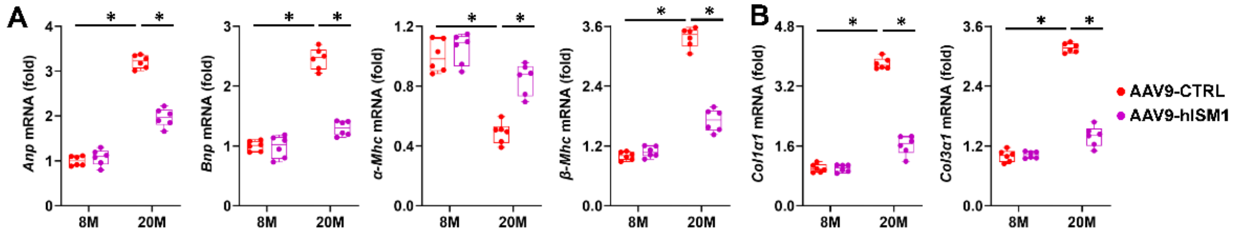

Supplementary Figure 3. ISM1 attenuates aging-related cardiac dysfunction and remodeling. A Relative *Anp*, *Bnp*, *α-Mhc*, and *β-Mhc* mRNA levels in hearts (n=6). **B** Relative *Col1 α 1 and Col3 α 1* mRNA levels in hearts (n=6). Comparisons between two groups were performed using an unpaired two-tailed Student's *t*-test, whereas one-way analysis of variance followed by Tukey post hoc test was conducted for comparisons among three or more groups. Values represent the mean ± SEM. *P < 0.05 versus the matched group.

© 2024. Hu M et al. Published online at http://www.aginganddisease.org/EN/10.14336/AD.2024.0113

SUPPLEMENTARY DATA

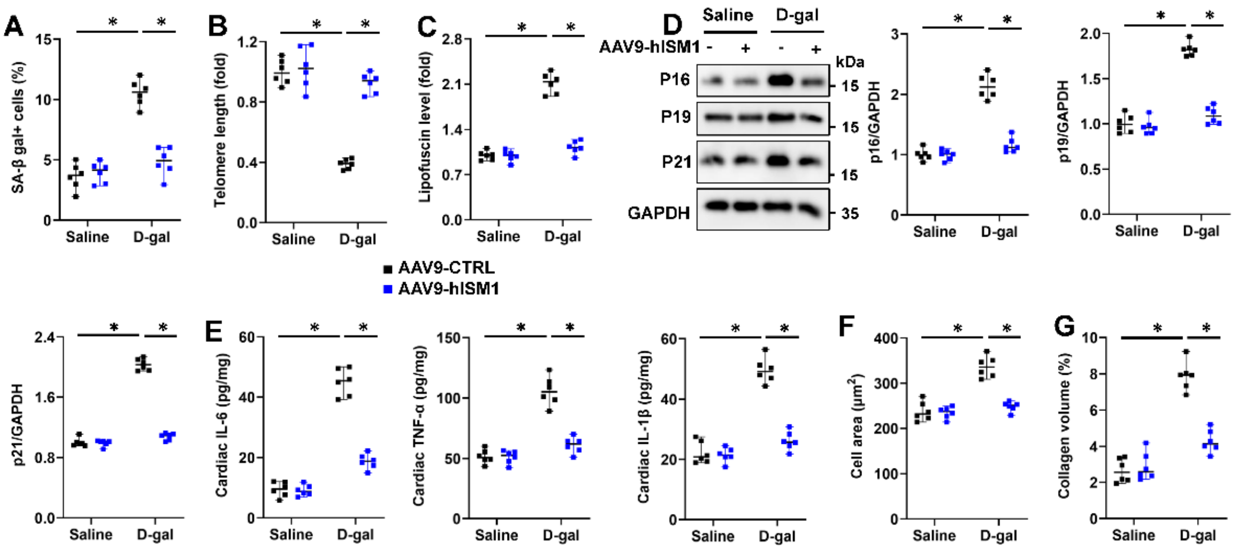

Supplementary Figure 4. ISM1 attenuates D-gal-induced cardiac aging. **A** Quantitative results of SA β-gal-stained cells (n=6). **B** Relative telomere length in murine hearts (n=6). **C** Cardiac lipofuscin content in murine hearts (n=6). **D** Representative western blot images and statistical results (n=6). **E** The myocardial IL-6, TNF-α and IL-1β levels were determined by ELISA kits (n=6). **F** Quantitative results of cardiomyocyte area (n=6). **G** Quantitative results of average collagen volume in mice (n=6). Comparisons between two groups were performed using an unpaired two-tailed Student's *t*-test, whereas one-way analysis of variance followed by Tukey post hoc test was conducted for comparisons among three or more groups. Values represent the mean ± SEM. *P < 0.05 versus the matched group.

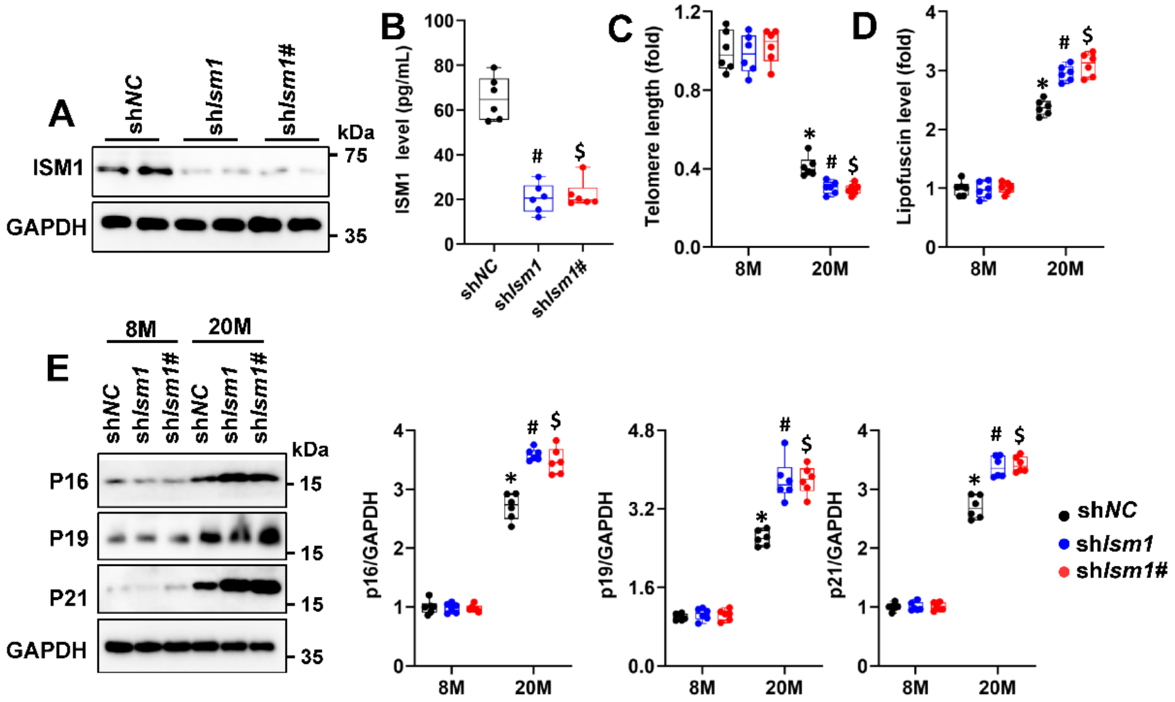

Supplementary Figure 5. ISM1 deficiency deteriorates aging-related cardiac aging. **A-B** Representative western blot images and statistical results (n=6). **C** Relative telomere length in murine hearts (n=6). **D** Cardiac lipofuscin content in murine hearts (n=6). **E** Representative western blot images and statistical results (n=6). Comparisons between two groups were performed using an unpaired two-tailed Student's *t*-test, whereas one-way analysis of variance followed by Tukey post hoc test was conducted for comparisons among three or more groups. Values represent the mean ± SEM. *P < 0.05 versus the matched group, # P < 0.05 sh*Ism1* versus sh*NC*, $ P < 0.05 sh*Ism1*# versus sh*NC*.

© 2024. Hu M et al. Published online at http://www.aginganddisease.org/EN/10.14336/AD.2024.0113

SUPPLEMENTARY DATA

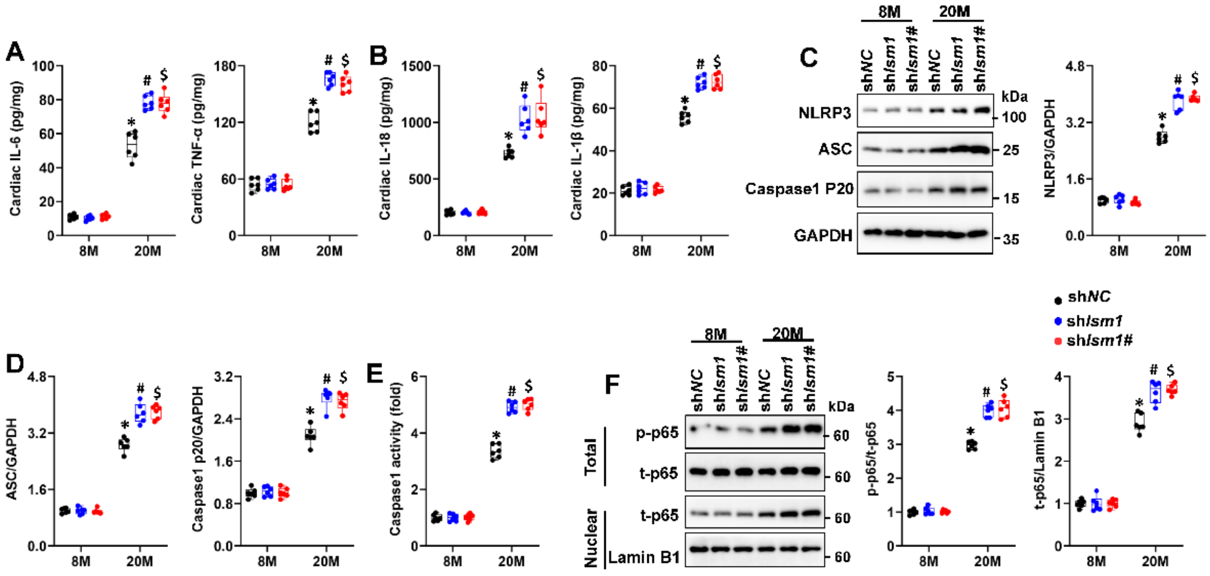

Supplementary Figure 6. ISM1 deficiency deteriorates aging-related inflammatory response in hearts. A The myocardial IL-6, TNF-α and IL-1β levels were determined by ELISA kits (n=6). **B** The myocardial IL-18 and IL-1β levels were determined by ELISA kits (n=6). C-D Representative western blot images and statistical results (n=6). **E** Caspase1 activity in hearts (n=6). **F** Representative western blot images and statistical results (n=6). Comparisons between two groups were performed using an unpaired two-tailed Student′s *t*-test, whereas one-way analysis of variance followed by Tukey post hoc test was conducted for comparisons among three or more groups. Values represent the mean ± SEM. *P < 0.05 versus the matched group, # P < 0.05 sh*Ism1* versus sh*NC*, $ P < 0.05 sh*Ism1#* versus sh*NC*.

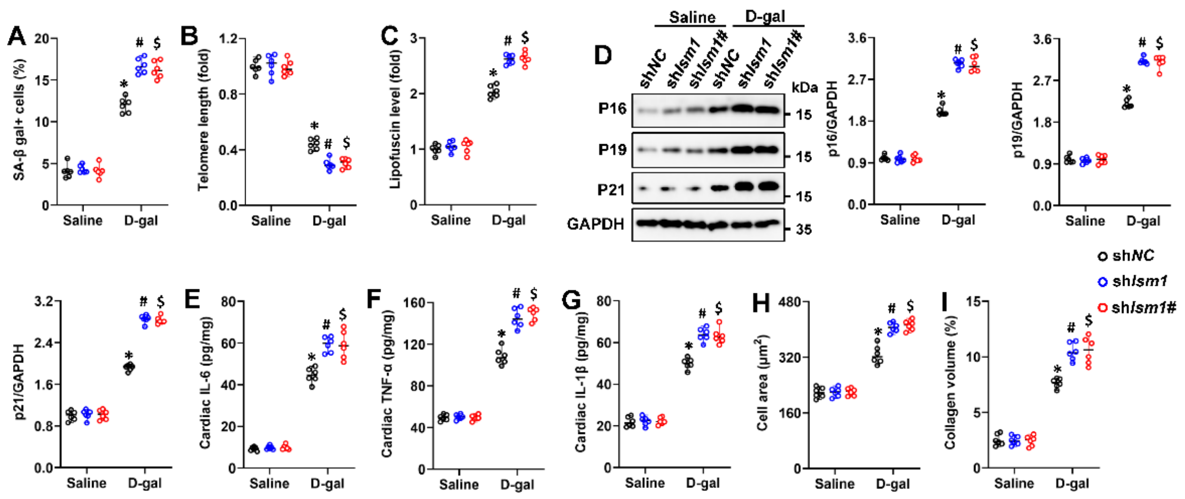

Supplementary Figure 7. ISM1 deficiency deteriorates D-gal-induced cardiac dysfunction. **A** Quantitative results of SA β-gal-stained cells (n=6). **B** Relative telomere length in murine hearts (n=6). **C** Cardiac lipofuscin content in murine hearts (n=6). **D** Representative western blot images and statistical results (n=6). E-G The myocardial IL-6, TNF-α and IL-1β levels were determined by ELISA kits (n=6). **H** Quantitative results of cardiomyocyte area (n=6). **I** Quantitative results of average collagen volume in mice (n=6). Comparisons between two groups were performed using an unpaired two-tailed Student′s *t*-test, whereas one-way analysis of variance followed by Tukey post hoc test was conducted for comparisons among three or more groups. Values represent the mean ± SEM. *P < 0.05 versus the matched group, # P < 0.05 sh*Ism1* versus sh*NC*, $ P < 0.05 sh*Ism1#* versus sh*NC*.

© 2024. Hu M et al. Published online at http://www.aginganddisease.org/EN/10.14336/AD.2024.0113

SUPPLEMENTARY DATA

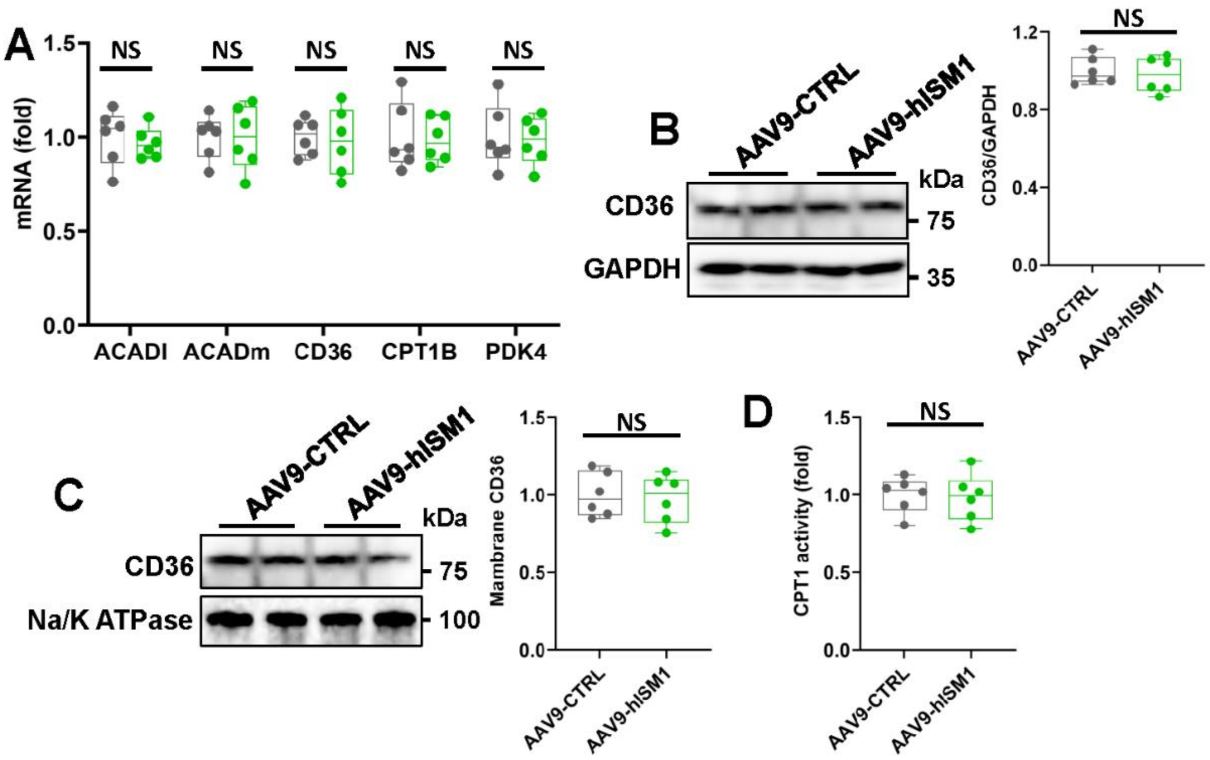

Supplementary Figure 8. ISM1 overexpression did not affect lipid metabolism. **A** Relative mRNA levels in hearts (n=6). **B** Representative western blot images and statistical results (n=6). **C** Representative western blot images and statistical results (n=6). **D** CPT1 activity in hearts (n=6). Comparisons between two groups were performed using an unpaired two-tailed Student′s *t*-test, whereas one-way analysis of variance followed by Tukey post hoc test was conducted for comparisons among three or more groups. Values represent the mean ± SEM. *P < 0.05 versus the matched group.

© 2024. Hu M et al. Published online at http://www.aginganddisease.org/EN/10.14336/AD.2024.0113

SUPPLEMENTARY DATA

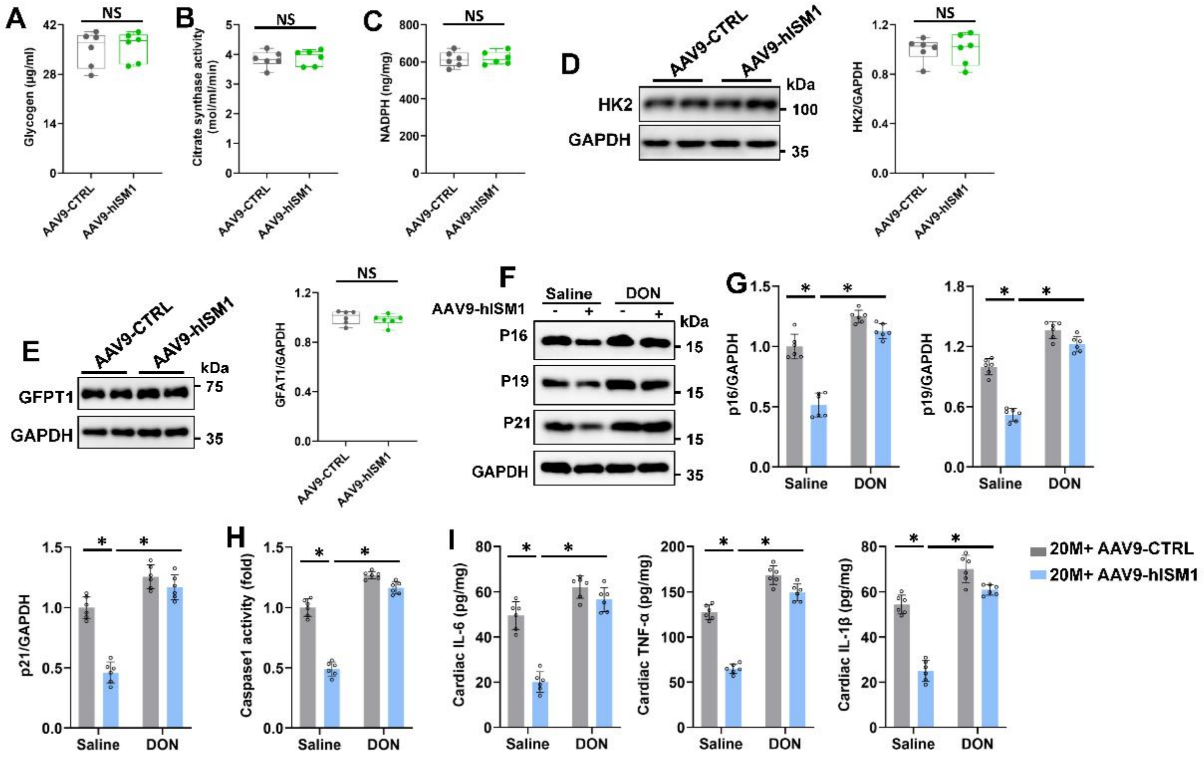

Supplementary Figure 9. DON treatment blunted the alleviation of ISM1 in aging mice. **A** Glycogen level in hearts (n=6). **B** Citrate synthase activity in hearts (n=6). **C** NADPH level in hearts (n=6). **D** Representative western blot images and statistical results (n=6). **E** Representative western blot images and statistical results (n=6). **F-G** Representative western blot images and statistical results (n=6). **H** Caspase1 activity in hearts (n=6). **I** The myocardial IL-6, TNF-α and IL-1β levels were determined by ELISA kits (n=6). Comparisons between two groups were performed using an unpaired two-tailed Student′s *t*-test, whereas one-way analysis of variance followed by Tukey post hoc test was conducted for comparisons among three or more groups. Values represent the mean ± SEM. *P < 0.05 versus the matched group.

© 2024. Hu M et al. Published online at http://www.aginganddisease.org/EN/10.14336/AD.2024.0113

SUPPLEMENTARY DATA

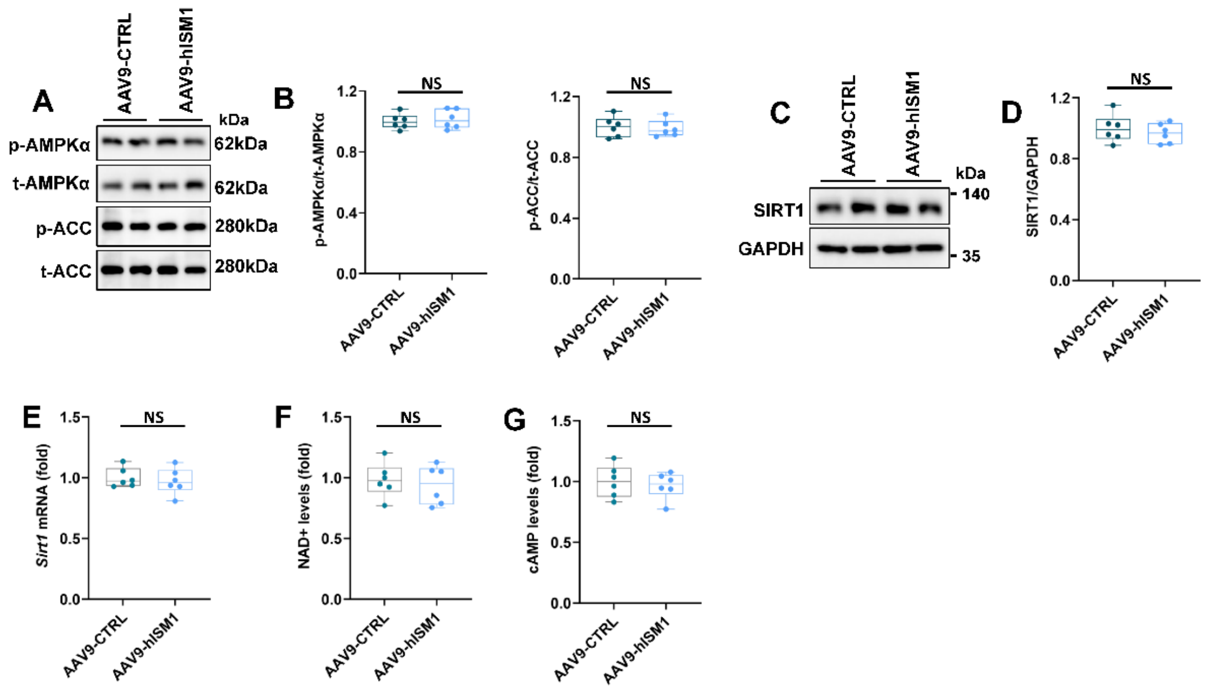

Supplementary Figure 10. ISM1 overexpression elevates SIRT1 deacetylase activity in aging hearts. A-B Representative western blot images and statistical results (n=6). **C-D** Representative western blot images and statistical results (n=6). **E** Relative *Sirt1* mRNA levels in hearts (n=6). **F** NAD+ level in hearts (n=6). **G** CAMP level in hearts (n=6). Comparisons between two groups were performed using an unpaired two-tailed Student's *t*-test, whereas one-way analysis of variance followed by Tukey post hoc test was conducted for comparisons among three or more groups. Values represent the mean ± SEM. *P < 0.05 versus the matched group.

© 2024. Hu M et al. Published online at http://www.aginganddisease.org/EN/10.14336/AD.2024.0113

SUPPLEMENTARY DATA

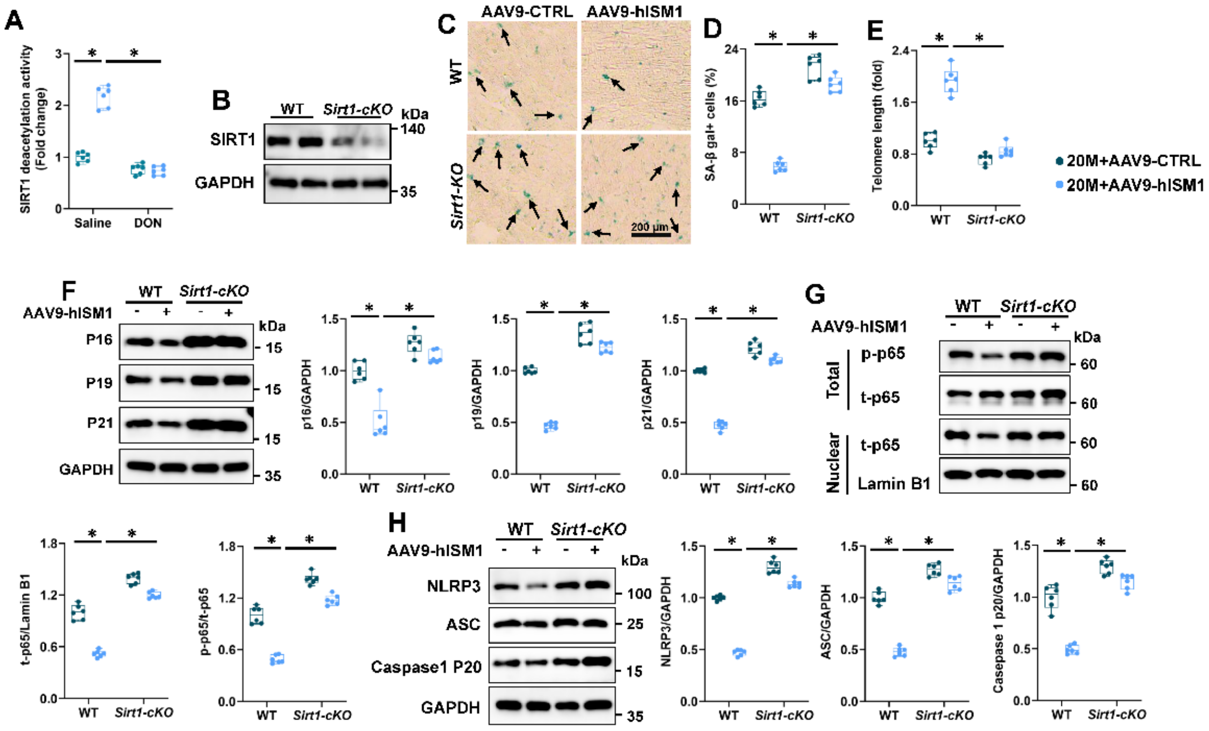

Supplementary Figure 11. ISM1 lost its protective effects against aging-related cardiac inflammation after SIRT1 deficiency. A SIRT1 deacetylase activity in hearts (n=6). **B** Representative western blot images (n=6). **C-D** Quantitative result of SA-β gal-stained heart sections (n=6). **E** Relative telomere length in murine hearts (n=6). **F** Representative western blot images and statistical results (n=6). **G** Representative western blot images and statistical results (n=6). **H** Representative western blot images and statistical results (n=6). Comparisons between two groups were performed using an unpaired two-tailed Student′s *t*-test, whereas one-way analysis of variance followed by Tukey post hoc test was conducted for comparisons among three or more groups. Values represent the mean ± SEM. *P < 0.05 versus the matched group.

© 2024. Hu M et al. Published online at http://www.aginganddisease.org/EN/10.14336/AD.2024.0113

SUPPLEMENTARY DATA

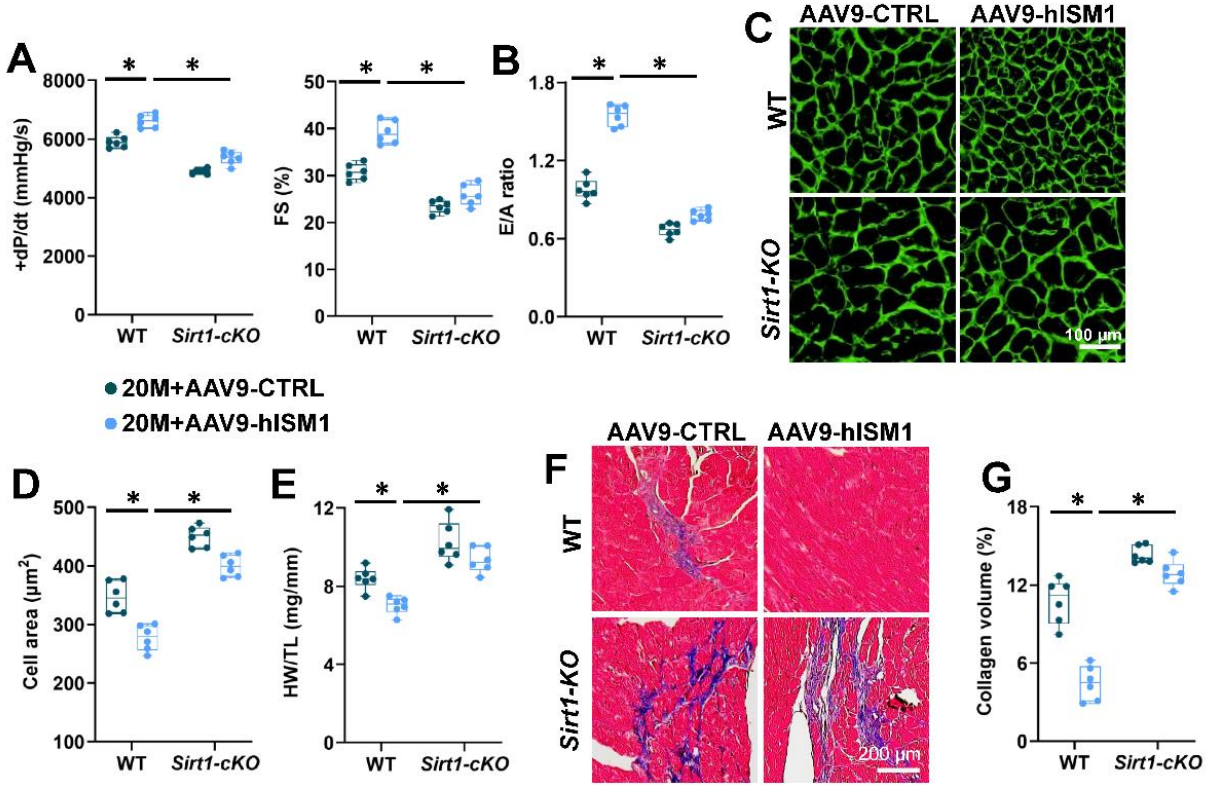

Supplementary Figure 12. ISM1 lost its protective effects against aging-related cardiac hypertrophy and fibrosis after SIRT1 deficiency. **A** +dP/dt and FS of mice were determined by echocardiography (n=6). **B** The ratio of E/A (n=6). **C-D** Representative image of WGA staining and quantitative results in heart sections (n=6). **E** HW/TL in mice (n=6). **F-G** Representative image of MASSON staining and quantitative results in heart sections (n=6). Comparisons between two groups were performed using an unpaired two-tailed Student′s t-test, whereas one-way analysis of variance followed by Tukey post hoc test was conducted for comparisons among three or more groups. Values represent the mean ± SEM. *P < 0.05 versus the matched group.

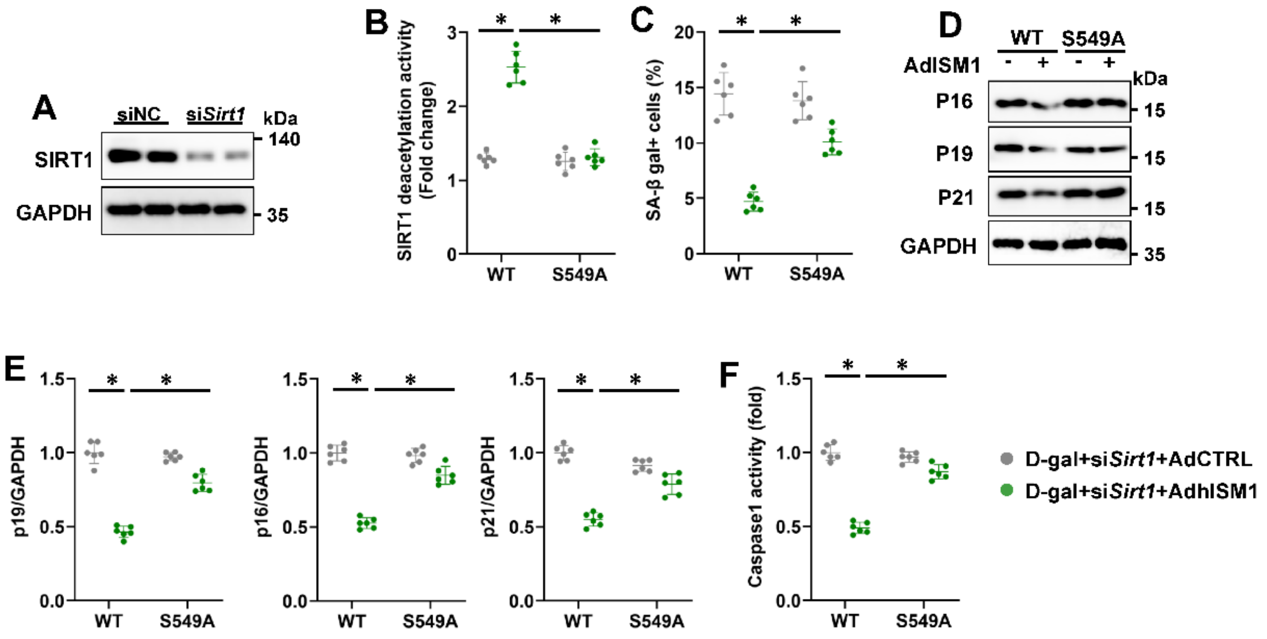

Supplementary Figure 13. SIRT1 is O-GlcNAcylated at Ser 549 in ISM1 overexpressed H9C2 cells. A Representative western blot images (n=6). **B** SIRT1 deacetylase activity in H9C2 cells (n=6). **C** Quantitative result of SA-β gal-stained cells (n=6). **D-E** Representative western blot images and statistical results (n=6). **F** Caspase1 activity in hearts (n=6). Comparisons between two groups were performed using an unpaired two-tailed Student's *t*-test, whereas one-way analysis of variance followed by Tukey post hoc test was conducted for comparisons among three or more groups. Values represent the mean ± SEM. *P < 0.05 versus the matched group.

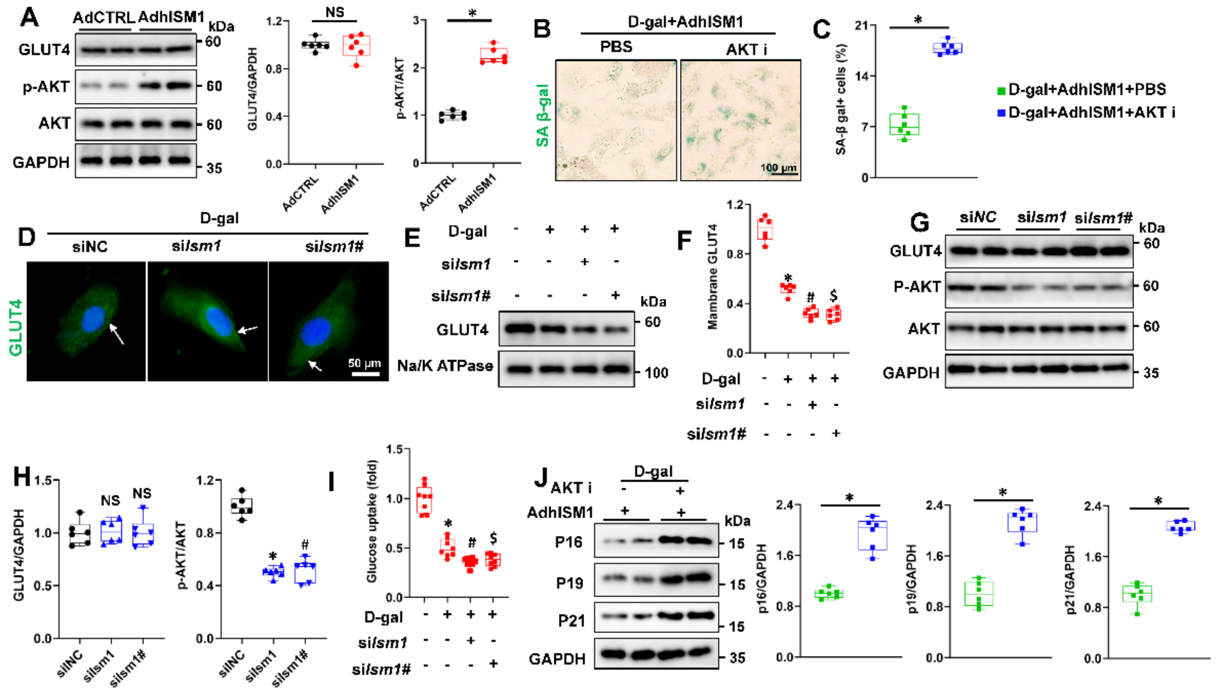

Supplementary Figure 14. ISM1 promotes glucose uptake in H9C2 via translocating GLUT4 to the cell surface. A Representative western blot images and statistical results (n=6). **B-C** Representative pictures of SA-β gal-stained heart sections and quantitative results

© 2024. Hu M et al. Published online at http://www.aginganddisease.org/EN/10.14336/AD.2024.0113

(n=6). **D** Representative image of GLUT4 staining in H9C2 (n=6). **E-F** Representative western blot images and statistical results (n=6). **G-H** Representative western blot images and statistical results (n=6). I Glucose uptake in H9C2 (n=8). **J** Representative western blot images and statistical results (n=6). Comparisons between two groups were performed using an unpaired two-tailed Student′s *t*-test, whereas one-way analysis of variance followed by Tukey post hoc test was conducted for comparisons among three or more groups. Values represent the mean ± SEM. *P < 0.05 versus the matched group.

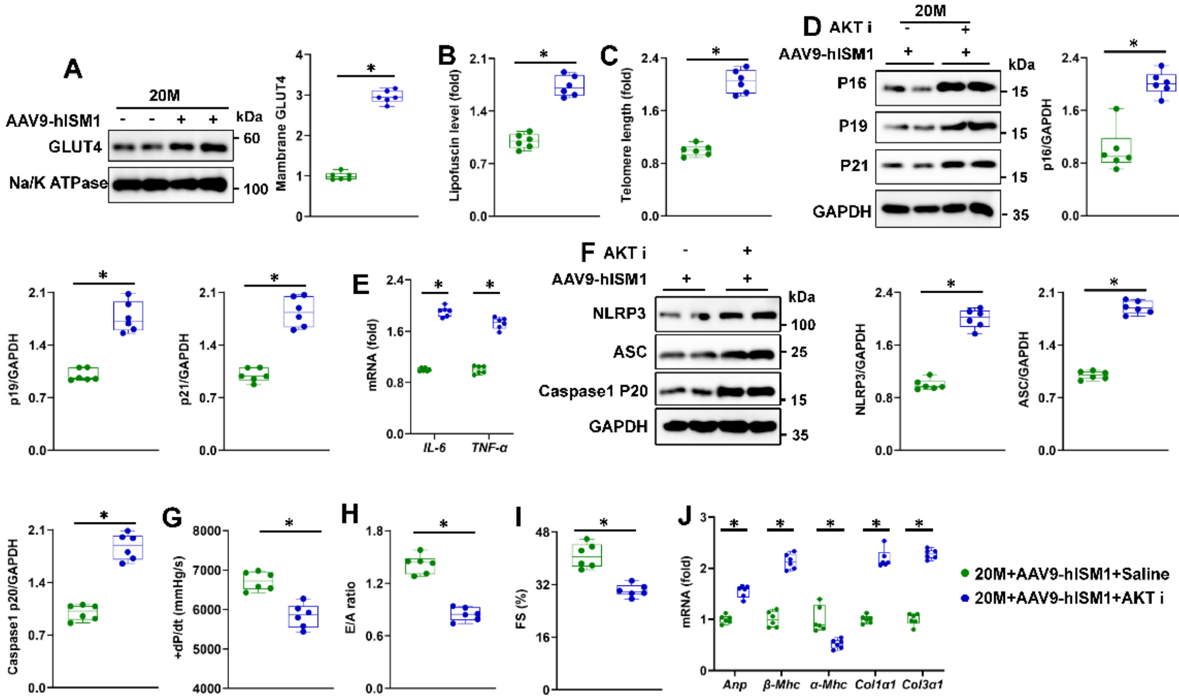

Supplementary Figure 15. ISM1 promotes glucose uptake in hearts via translocating GLUT4 to the cell surface. A Representative western blot images and statistical results (n=6). **B** Cardiac lipofuscin content in murine hearts (n=6). **C** Relative telomere length in murine hearts (n=6). **D** Representative western blot images and statistical results (n=6). **E** Relative *Il-6*, *Bnp* and *Tnf-α*mRNA levels in hearts (n=6). **F** Representative western blot images and statistical results (n=6). G +dP/dt of mice was determined by echocardiography (n=6). **H** The ratio of E/A (n=6). **I** FS in mice (n=6). J A Relative *Anp*, *α-Mhc*, *β-Mhc*, *Col1α1 and Col3α1*mRNA levels in hearts (n=6). Comparisons between two groups were performed using an unpaired two-tailed Student′s *t*-test, whereas one-way analysis of variance followed by Tukey post hoc test was conducted for comparisons among three or more groups. Values represent the mean ± SEM. *P < 0.05 versus the matched group.

© 2024. Hu M et al. Published online at http://www.aginganddisease.org/EN/10.14336/AD.2024.0113

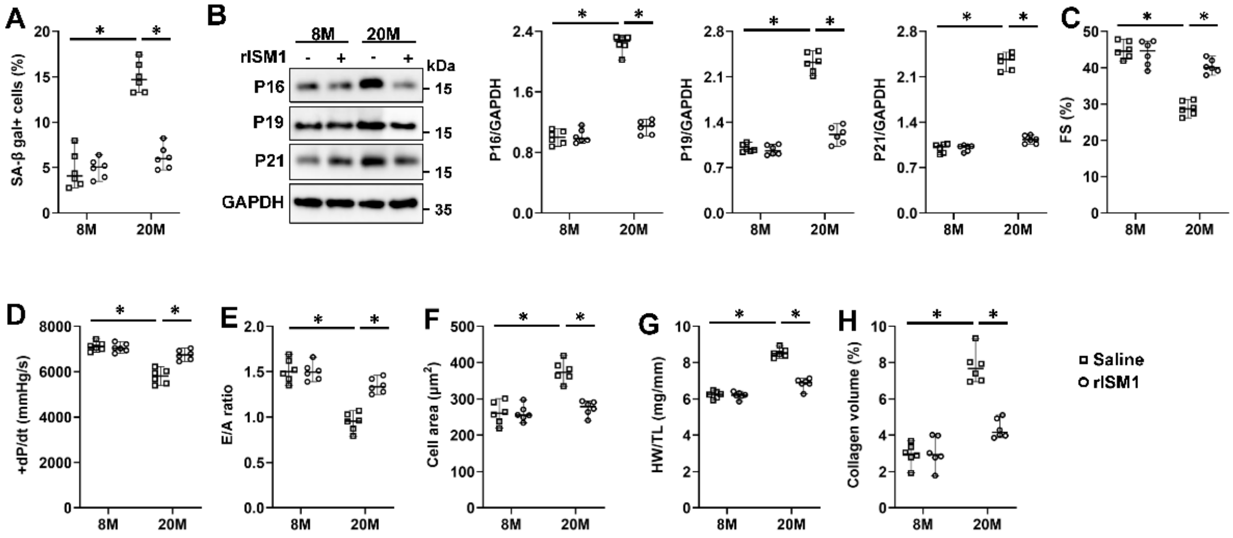

Supplementary Figure 16. RISM1 infusion mitigates aging-related cardiac dysfunction in vivo. **A** Quantitative results of SA β-gal-stained cells (n=6). **B** Representative western blot images and statistical results (n=6). **C** FS in mice (n=6). D +dP/dt of mice was determined by echocardiography (n=6). **E** The ratio of E/A (n=6). **F** Quantitative result of cardiomyocyte area in mice (n=6). **G** HW/TL in mice (n=6). **H** Quantitative result of average collagen volume (n=6). Comparisons between two groups were performed using an unpaired two-tailed Student′s *t*-test, whereas one-way analysis of variance followed by Tukey post hoc test was conducted for comparisons among three or more groups. Values represent the mean ± SEM. *P < 0.05 versus the matched group.

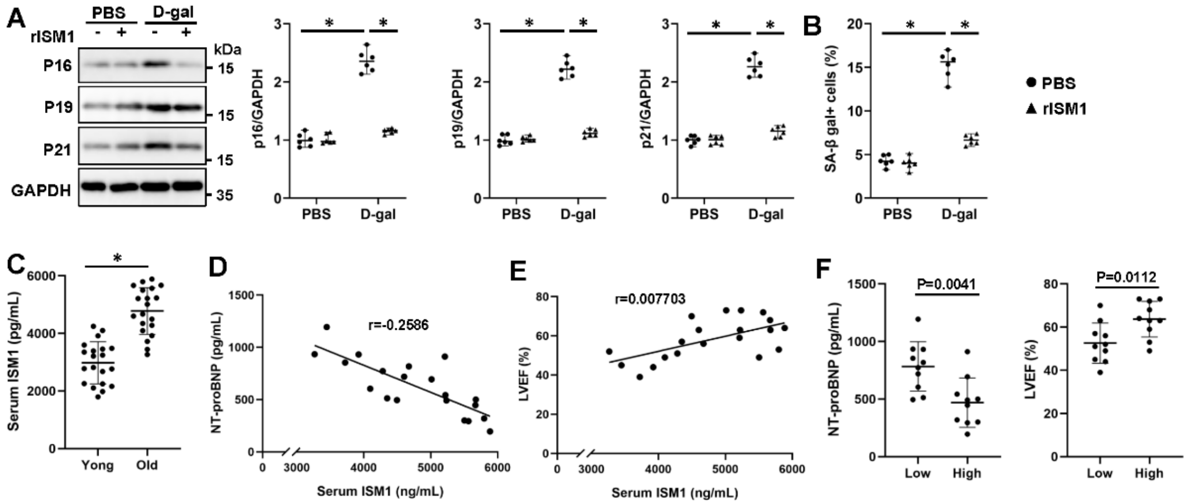

Supplementary Figure 17. RISM1 infusion mitigates D-gal-induced cellar senescence in vitro. **A** Representative western blot images and statistical results (n=6). **B** Quantitative results of SA β-gal-stained cells (n=6). **C** Serum ISM1 level (n=20). **D-E** Pearson linear correlation analysis between serum ISM1 and NT-ProBNP and LVEF (n=20). **F** Serum cTnI levels and LVEF (n=10). Comparisons between two groups were performed using an unpaired two-tailed Student′s *t*-test, whereas one-way analysis of variance followed by Tukey post hoc test was conducted for comparisons among three or more groups. Values represent the mean ± SEM. *P < 0.05 versus the matched group.

© 2024. Hu M et al. Published online at http://www.aginganddisease.org/EN/10.14336/AD.2024.0113
